# Polygenic Analysis of Tolerance to Carbon Dioxide Inhibition of Isoamyl Acetate “Banana” Flavor Production in Yeast Reveals *MDS3* as Major Causative Gene

**DOI:** 10.1128/aem.00814-22

**Published:** 2022-09-08

**Authors:** Ben Souffriau, Sylvester Holt, Arne Hagman, Stijn De Graeve, Philippe Malcorps, Maria R. Foulquié-Moreno, Johan M. Thevelein

**Affiliations:** a Laboratory of Molecular Cell Biology, Institute of Botany and Microbiology, KU Leuven, Leuven, Belgium; b Center for Microbiology, VIB, Leuven-Heverlee, Flanders, Belgium; c AB-InBev SA/NV, Leuven, Belgium; Royal Botanic Gardens

**Keywords:** QTL analysis, flavor production, isoamyl acetate, isoamyl alcohol, acetate esters, carbon dioxide, alcohol acetyl transferase, yeast

## Abstract

The introduction in modern breweries of tall cylindroconical fermentors, replacing the traditional open fermentation vats, unexpectedly revealed strong inhibition of flavor production by the high CO_2_ pressure in the fermentors. We have screened our collection of Saccharomyces cerevisiae strains for strains displaying elevated tolerance to inhibition of flavor production by +0.65 bar CO_2_, using a laboratory scale CO_2_ pressurized fermentation system. We focused on the production of isoamyl acetate, a highly desirable flavor compound conferring fruity banana flavor in beer and other alcoholic beverages, from its precursor isoamyl alcohol (IAAc/Alc ratio). We selected the most tolerant Saccharomyces cerevisiae strain, saké yeast Kyokai no. 1, isolated a stable haploid segregant seg63 with the same high IAAc/Alc ratio under CO_2_ pressure, crossed seg63 with the unrelated inferior strain ER7A and phenotyped 185 haploid segregants, of which 28 displaying a high IAAc/Alc ratio were pooled. Mapping of Quantitative Trait Loci (QTLs) by whole-genome sequence analysis based on SNP variant frequency revealed two QTLs. In the major QTL, reciprocal hemizygosity analysis identified *MDS3* as the causative mutant gene, a putative member of the TOR signaling pathway. The *MDS3*^Seg.63^ allele was dominant and contained a single causative point mutation, T2171C, resulting in the F274S substitution. Introduction of *MDS3*^Seg.63^ in an industrial tetraploid lager yeast with CRISPR/Cas9 enhanced isoamyl acetate production by 145% under CO_2_ pressure. This work shows the strong potential of polygenic analysis and targeted genetic modification for creation of cisgenic industrial brewer's yeast strains with specifically improved traits.

**IMPORTANCE** The upscaling of fermentation to very tall cylindroconical tanks is known to negatively impact beer flavor. Most notably, the increased CO_2_ pressure in such tanks compromises production by the yeast of the desirable fruity “banana” flavor (isoamyl acetate). The cause of the CO_2_ inhibition of yeast flavor production has always remained enigmatic. Our work has brought the first insight into its molecular-genetic basis and provides a specific gene tool for yeast strain improvement. We first identified a yeast strain with superior tolerance to CO_2_ inhibition of flavor production, and applied polygenic analysis to identify the responsible gene. We narrowed down the causative element to a single nucleotide difference, *MDS3*^T2171C^, and showed that it can be engineered into brewing yeast to obtain strains with superior flavor production in high CO_2_ pressure conditions, apparently without affecting other traits relevant for beer brewing. Alternatively, such a strain could be obtained through marker-assisted breeding.

## INTRODUCTION

As a result of industrialization and company growth, beer breweries began to brew continuously larger quantities of beer. This has prompted a shift from horizontal open vessels to deep, vertical cylindroconical tanks used for yeast fermentation at large commercial scale. Unexpectedly, this new fermentor design resulted in compromised yeast growth, sluggish fermentation, poor diacetyl stripping and insufficient fruity flavors in the beer (1–8). The latter is caused by inadequate ester production by the yeast, mainly of isoamyl acetate, a key aroma compound responsible for the fruity “banana” flavor of beer. The main inhibiting agent of isoamyl acetate productivity turned out to be the high level of dissolved CO_2_ in the cylindroconical tanks, which increases proportionally with the hydrostatic pressure at increasing depths of the fermentor (1). Large-scale beer production is performed in cylindroconical tanks with depths reaching 10 to 18 m, leading to hydrostatic pressures of approximately 1.0–1.8 bar (approximately 1.0–1.8 atmospheric pressure units). When yeast is subjected to CO_2_ pressure during alcoholic fermentation the formation of fusel alcohols and acetate esters is strongly inhibited (4, 9). At CO_2_ pressures above 0.5 bar, the exponential growth rate starts to be reduced, with complete inhibition at 2.7 bar (10). A CO_2_ overpressure of 1 bar corresponds approximately to the hydrostatic pressure at 10 m depth, and causes a drop in isoamyl acetate production from 3.6 to 1.4 mg/L, which compromises beer quality (11).

Isoamyl acetate is uniquely produced by the yeast alcohol acetyl coenzyme A (acetyl-CoA) transferase enzymes (AATases), Atf1 and Atf2, by condensation of the precursor molecules isoamyl alcohol and acetyl-CoA (12). High CO_2_ pressure not only inhibits AATase activity, but also formation of the precursors isoamyl alcohol and other fusel alcohols (3). The ratio between isoamyl acetate and isoamyl alcohol (IAAc/Alc ratio) is therefore a more reliable readout for the AATase activity and its sensitivity to high CO_2_ pressure. It is clear that Atf1 is responsible for the majority of AATase activity for production of the flavor-active acetate esters, with isoamyl acetate levels being more than 80% reduced by *ATF1* deletion and 180-fold increased by constitutive overexpression of *ATF1* in a laboratory yeast (13). The major limiting factor for production of isoamyl acetate is the expression level of *ATF1*, which correlates with the final concentration of isoamyl acetate in beer (13–15). Acetate ester production by Atf1 is regulated by a number of factors. It is inhibited by dissolved oxygen and low nitrogen content, and enhanced by high gravity (i.e., high sugar level), whereas the effects of fermentation temperature and pitching rate appear to be strain-dependent (12).

Fujiwara et al. (16) have performed molecular characterization of the *ATF1* promoter elements and showed that activation by the Rap1 repressor/activator transcription factor is essential for *ATF1* expression. Rap1 has roles in many cellular responses, including induction of ribosomal genes during growth (17). Recently, a truncated allele of the Tor1 kinase was shown to enhance the levels of acetate esters in alcoholic fermentations (18). TOR (Target Of Rapamycin) consists of the Tor1 and Tor2 kinases, which form the TORC1 and TORC2 complexes, that regulate various cellular functions. TORC1 contains either Tor1 or Tor2, and TORC2 exclusively Tor2. Disruption of *TOR1* only affects the growth of yeast, whereas *TOR2* is an essential gene for yeast survival (19). TORC1 functions in a major growth promoting signal transduction pathway and acts as regulator of nitrogen catabolite repression genes (20). Fujiwara et al. (16) found that *ATF1* expression is hampered by deletion of the downstream TORC1 effector kinase Sch9, linking the TORC1 pathway directly to *ATF1* expression. On the other hand, it has also been shown that high activity of the protein kinase A (PKA) signaling pathway, another major growth activator, increases the transcript level of the *ATF1* gene (21).

On the other hand, the growth inhibitory effects of CO_2_ are thought to act through direct inhibition of metabolic enzymes and membrane integrity, as well as lowered intracellular pH (bicarbonate) and mitochondrial function (10, 22–24). The uptake of branched-chain amino acids (precursors of fusel alcohols and esters) is inhibited, cells are enlarged in size and viability drops (3, 25). Hence, the inhibition of AATase activity by high CO_2_ pressure may be caused by different mechanisms and/or physiological stress responses. The relevance of the different mechanisms proposed remains unclear.

In recent years, technological advances in next-generation whole-genome sequencing and in genome editing using CRISPR/Cas9, have facilitated the identification of single point mutations responsible for quantitative traits. The sexual life cycle and tractability of the yeast Saccharomyces cerevisiae has made it the preferred organism to map quantitative trait loci (QTLs) with significant linkage disequilibrium in the genome in selected segregant populations. Pooled-segregant QTL analysis is now well established as a powerful tool to elucidate the genetic basis of complex yeast traits in which multiple unknown genetic loci are involved, and which are important in industrial applications (26–28). Examples of polygenic analysis for the latter with respect to flavor-active metabolite production include altered production of the acetate esters ethyl acetate (“solvent-like” descriptor) and phenyl ethyl acetate (“rose” and “honey” descriptors), ethyl esters (“apple” descriptor), fusel alcohols (“alcoholic” and “vinous” descriptors) and the polyfunctional thiol 4-sulfanyl-4-methylpentan-2-one (“grape fruit” and “cats pee” descriptors), acetic acid (“vinegar” descriptor), sulfite and hydrogen disulphide (“rotten eggs” descriptor) (18, 29–35).

**FIG 1:**
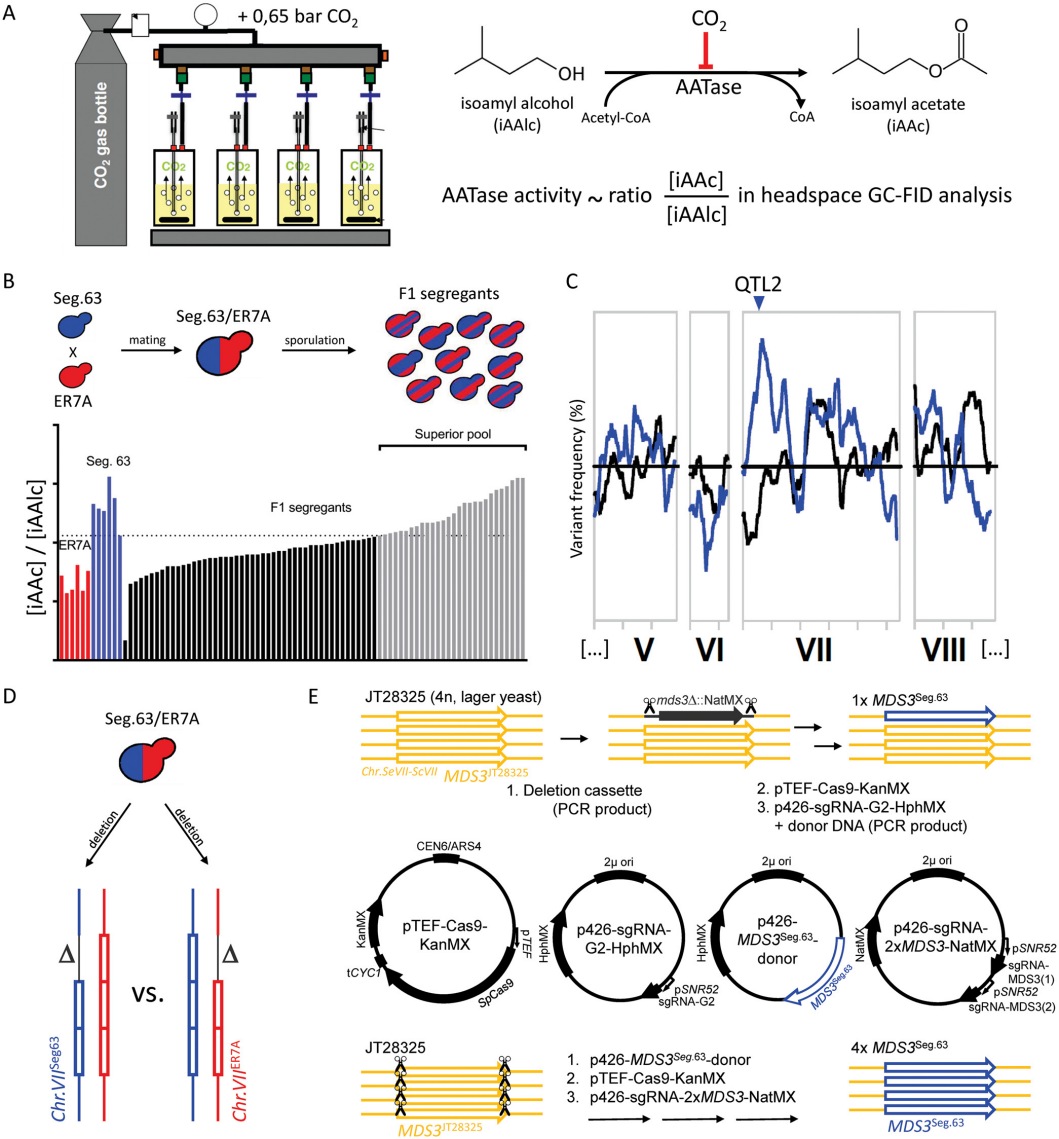
Overview of experimental methods used in this study. (A) Lab-scale determination of the CO_2_ overpressure inhibition of acetate ester production (isoamyl acetate/alcohol ~AATase activity). (B) Mating and breeding segregants (offspring) with a range of CO_2_-inhibition of AATase activity, followed by selection of a pool of segregants with a superior acetate ester production profile. (C) Whole-genome sequence analysis of the superior pool and bioinformatic analysis to identify QTLs responsible for the trait. (D) Schematic representation of (Bulk) RHA as used to identify the causative gene in QTL 2. (E) Graphical overview of the 2 strategies and different plasmids used for CRISPR/Cas9 mediated *MDS3* allele exchange.

We have applied the polygenic analysis platform for identification of mutant alleles conferring superior tolerance to CO_2_ inhibition of the production of the crucial flavor compound isoamyl acetate. For that purpose, we identified in our S. cerevisiae strain collection strains displaying superior tolerance and performed QTL mapping by pooled-segregant whole-genome sequence analysis, which revealed a single major QTL with *MDS3* as causative gene. The superior allele of the *MDS3* gene contained a unique causative SNP, which conferred the majority of the high tolerance to CO_2_ inhibition of isoamyl acetate production in the original genetic background. The superior *MDS3* allele also improved the same trait in an S. pastorianus lager brewing yeast strain without significantly affecting the production of other flavor compounds. Fig. 1 shows a graphical overview of the experimental methods used in this study.

## RESULTS

### Methodology for evaluation of tolerance of the IAAc/Alc ratio to high CO_2_ pressure in small-scale fermentations.

**FIG 2:**
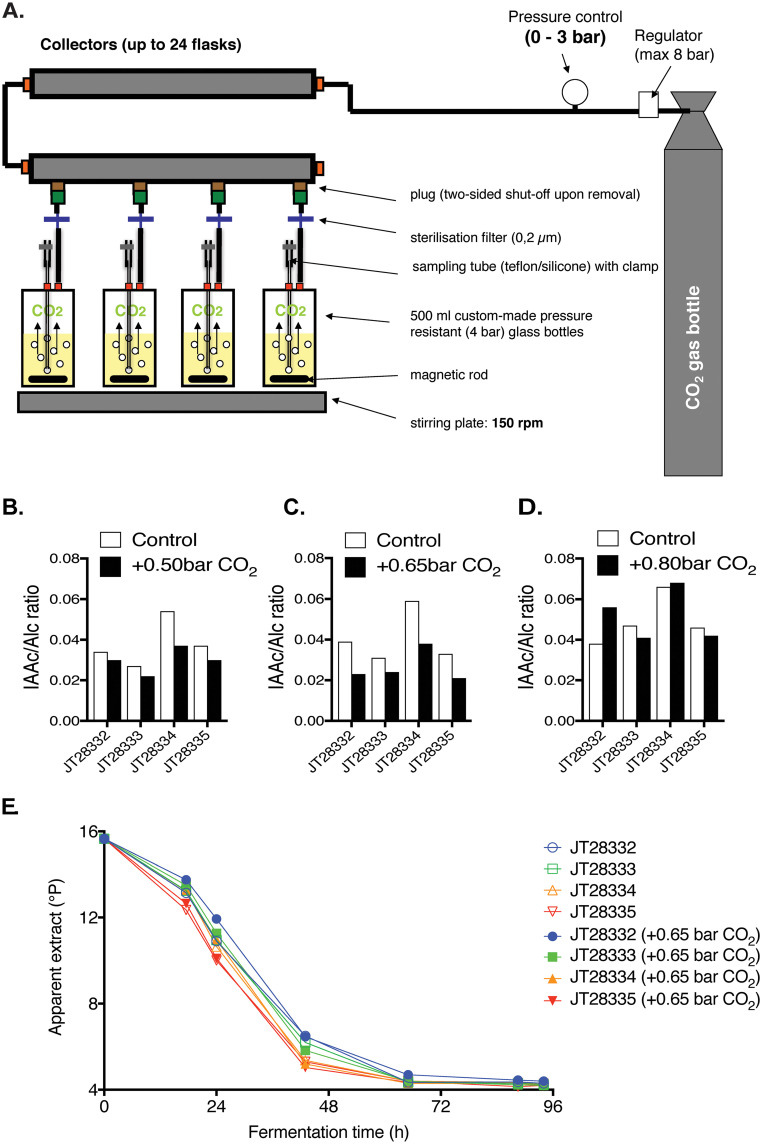
The lab-scale set-up for high CO_2_ pressure fermentation. (A) Scheme of the lab-scale high CO_2_ pressure fermentation system. (B) (C) (D) IAAc/Alc ratio after fermentation with 4 brewing yeast strains with and without extra CO_2_ pressure: (B). +0.50 bar, (C). +0.65 bar, and (D). +0.80 bar (E). Fermentation progress (apparent extract, in °Plato) with and without extra CO_2_ pressure (+0.65 bar). Strain JT28332 is an ale yeast and strains JT28333 to JT28335 are Frohberg lager yeasts.

To mimick the high CO_2_ pressure in large-scale industrial brewing fermentors, we designed a laboratory scale fermentation system with increased CO_2_ pressure applied from a gas bottle (Fig. 2A). The system consisted of a gas bottle with an initial pressure regulator up to 12 bar, connected via pressure resistant tubing to a more precise pressure control unit operating between 0 and 3 bar. From there, the gas flows to collectors in which 10 removable plugs can be fitted. These plugs are connected to 0.5L pressure resistant fermentation bottles (up to 4 bar) through tubing with a filter for sterilization of the gas flow and a safety valve for release of excess pressure (> 1 bar). The fermentation bottles are equipped with a sampling valve and placed on electromagnetic stirring plates for continuous stirring.

Using the lab-scale fermentation system with simulated CO_2_ pressure, we first evaluated the effect of 0.50–1.50 bar CO_2_ overpressure on the AATase activity of 4 representative brewing yeasts by quantification of the IAAc/Alc ratio at the end of the beer fermentations. As expected, the AATase activity was significantly inhibited by CO_2_ overpressure of 0.50 and 0.65 bar in a strain specific manner, with the strongest inhibition observed for the JT28334 Frohberg lager yeast (Fig. 2B and C). Unexpectedly, the higher reduction of isoamyl alcohol levels at 0.80 bar compared to that at lower pressures was not accompanied by a stronger reduction in the isoamyl acetate levels, causing IAAc/Alc ratios to be similar to those in non-pressurized conditions (Fig. 2D). Moreover, we attempted to gain maximal inhibition of AATase activity by applying 1.50 bar overpressure after an initial growth and fermentation phase of 30h at 0.50 bar, but similarly the AATase activity (IAAc/Alc ratio) did not appear to be decreased further (data not shown). Apart from hydrostatic pressure, ester, and higher alcohol production is also linked to yeast growth. At low pressure values, yeast growth remains unaffected. If a high pressure is applied, like 0.8 bar in Fig. 2D, yeast growth becomes affected and therefore other effects start to play that are beyond the regulation of the alcohol to ester reaction. Different strains show different sensitivities in terms of growth inhibition. These effects may be related to the intracellular acetyl-CoA pool required for ester formation, even though we have not investigated this route. The final assay used for all subsequent pressurized fermentations applied 0.65 bar CO_2_ overpressure, which caused a clear inhibition of AATase activity and only minimal inhibition of fermentation activity (Fig. 2E).

### Screening of strains for tolerance of the IAAc/Alc ratio to high CO_2_ pressure.

**FIG 3:**
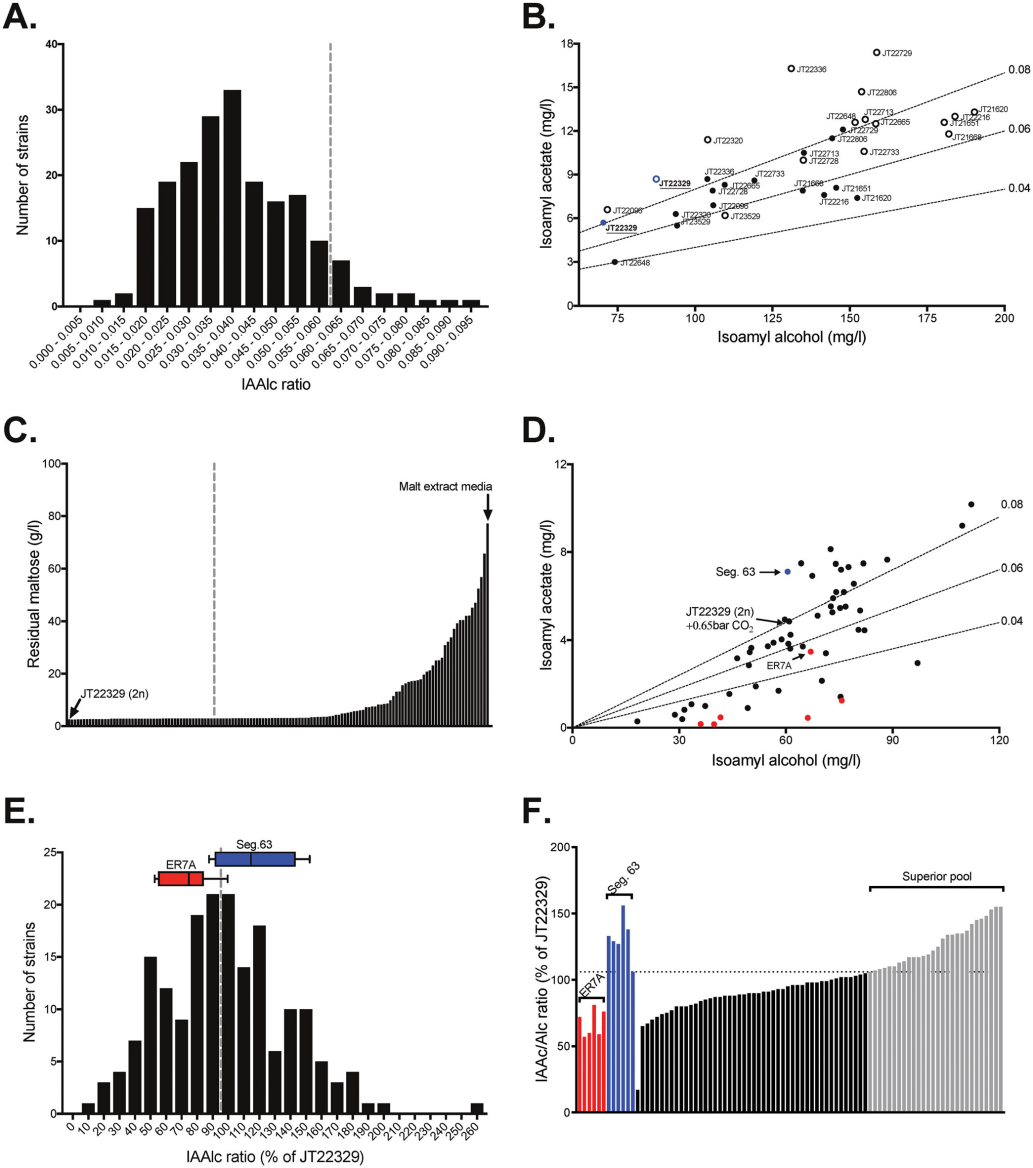
Screening for yeast strains producing superior IAAc/Alc ratios in high CO_2_ pressure fermentations. (A) Frequency distribution of the IAAc/Alc ratio in 200 preselected *Saccharomyces* strains obtained with gas chromatography analysis of fermentations in malt extract media under 0.65 bar extra CO_2_ pressure. The broken gray line indicates the cut-off for the 16 strains with the highest IAAc/Alc ratio (>0.06). (B) Scatterplot of isoamyl acetate and isoamyl alcohol levels produced in fermentations of malt extract medium, showing the resilience to extra CO_2_ pressure of the top 16 strains with the highest IAAc/Alc ratio. Filled circles indicate fermentations with 0.65 bar extra CO_2_ pressure and empty circles indicate fermentations without extra CO_2_ pressure. Dotted lines represent the average IAAc/Alc ratios (0.04, 0.06 and 0.08). The symbols of the selected superior strain, JT22329 (Kyokai no. 1) are shown in blue. (C) Residual maltose level after 4 days of fermentation in malt extract medium (15°P, non-aerated) for 120 segregants of strain JT22329 (Kyokai no. 1). The broken gray line indicates the 49 segregants with the lowest residual maltose level that were subsequently subjected to flavor profiling in pressurized beer fermentations. (D) Scatterplot of isoamyl acetate and isoamyl alcohol levels produced in 0.65 bar pressurized beer fermentations with maltose fermenting segregants of JT22329. Dotted lines represent the average IAAc/Alc ratios (0.04, 0.06 and 0.08). The symbol of the selected superior segregant is shown in blue and symbols of unrelated inferior haploid strains are indicated in red. Arrows indicate the selected superior segregant (Seg.63), the unrelated inferior haploid strain (ER7A) selected for mating, and the diploid parental strain (JT22329) for fermentations with and without extra CO_2_ pressure. (E) Frequency distribution of IAAc/Alc ratios obtained from gas chromatography analysis of fermentations in malt extract medium with 0.65 bar extra CO_2_ pressure for 185 haploid segregants of the hybrid diploid Seg.63/ER7A preselected for efficient maltose fermentation. The ratios have been normalized per fermentation batch to that of strain JT22329. The haploid parental strains, Seg.63 and ER7A, were included in each of the eight fermentation batches, and are shown on top with 10–90 percentile box plots. The broken gray line indicates the 100% cut-off for the 76 segregants selected for confirmation. (F) Confirmation and selection of the haploid segregants of Seg.63/ER7A showing the highest IAAc/Alc ratio. The ratios have been normalized per fermentation batch to that of strain JT22329. The dotted gray line indicates the 106% cut-off for the 28 segregants with the highest IAAc/Alc ratio, selected for the superior pool (shown in blue). The haploid parental strains, ER7A and Seg.63, included in each of the 6 batches, are shown in blue and red, respectively.

Due to the labor-intensive set-up of the CO_2_ pressurized fermentations, we first made a preselection based on high isoamyl acetate production by screening 423 Saccharomyces spp. strains in semi-anaerobic non-pressurized fermentations with yeast extract-peptone medium adjusted to a free amino nitrogen concentration of 250 mg/L and containing 10% (wt/vol) glucose. From this prescreening, we selected the 100 strains with the highest isoamyl acetate production (Fig. S1) for evaluation in pressurized fermentation conditions. This selection was expanded with 117 additional, previously tested strains (35), mainly coming from food applications, predominantly beer and wine. All these 217 strains showed complete maltose fermentation capacity within maximum 6 days in malt extract wort and were subjected to screening for superior CO_2_-resilient AATase activity in fermentations with oxygenated malt extract medium under 0.65 bar CO_2_ overpressure. When the fermentations were completed, we determined the metabolite profile with gas chromatography and derived the IAAc/Alc ratio as an indirect measure of the AATase activity. The results indicated that the IAAc/Alc ratio obtained for fermentations under inhibition by CO_2_ was log-normally distributed with a tailing at the higher ratios (Fig. 3A). The 16 strains with an IAAc/Alc ratio of at least 0.06 were evaluated again in fermentations with and without CO_2_ pressure to identify strains that maintained a high ratio in both conditions. Only 3 strains maintained an IAAc/Alc ratio over 0.08 both with and without CO_2_ pressure, out of which the Saké strain Kyokai no. 1 (JT22329) showed the lowest production of isoamyl alcohol and minimal inhibition of AATase activity by high CO_2_ pressure (Fig. 3B). This strain was therefore chosen for polygenic analysis of the underlying genetic elements.

### Selection of superior haploid segregant from superior diploid Kyokai no. 1.

Next, we deleted the 2 copies of the HO endonuclease gene in the diploid strain Kyokai no. 1 (JT22329) and sporulated the strain on minimal medium to obtain stable haploid spores. Although the parental strain was able to grow as well as ferment maltose, we observed segregation of the ability to ferment maltose in the haploid descendants. We therefore performed prescreening of 137 segregants for maltose fermentation in 24-well microtiter plates (Fig. 3C), and selected the 49 segregants that were able to fully complete the fermentation with malt extract. These were then evaluated in CO_2_ pressurized fermentations, which revealed segregants that performed even better than the parental strain Kyokai no. 1 (JT22329). The superior segregant 63 ('Seg.63') was one of the best performing strains (Fig. 3D). We included in this evaluation several unrelated haploid strains for possible use as reference inferior strain in the polygenic analysis and finally selected the ER7A strain, a segregant from the Ethanol Red bioethanol production strain, because it displayed an intermediate IAAc/Alc ratio.

### Assembly of a pool of superior segregants obtained from the hybrid diploid strain Seg.63/ER7A.

We have mated Seg.63 with ER7A, sporulated the hybrid diploid Seg.63/ER7A and obtained haploid offspring with a range of IAAc/Alc ratios. Again, we observed a segregation in maltose fermentation capacity with 65% of the 428 isolated segregants showing complete maltose fermentation after 4 days in malt extract wort in microtiter plates, whereas the remainder showed incomplete fermentation of the maltose (Fig. S2). All segregants were tested also for mating type with mating type specific PCR. The ploidy of the haploid segregants was confirmed by mating type PCR. Of the 279 maltose positive segregants, we screened 185 for strains displaying a high IAAc/Alc ratio under CO_2_ pressurized conditions. The data were normalized against the IAAc/Alc ratio of the Kyokai no. 1 (JT22329) parental strain, which was included as a control in every batch of fermentations, to account for batch to batch variation. Segregation of the IAAc/Alc ratio in the progeny was normally distributed with a median of 91% of the IAAc/Alc ratio of the Kyokai no. 1 (JT22329) strain, and ranging between 75% of the IAAc/Alc ratio in the inferior ER7A strain and 115% of that in the superior Seg.63 strain (Fig. 3E). We selected 76 segregants with an IAAc/Alc ratio at least as high as that of Kyokai no. 1 (JT22329) for further evaluation and identified 28 segregants with an IAAc/Alc ratio at least 106% of that of Kyokai no. 1 (JT22329), which we used to compose the superior pool for polygenic analysis (Fig. 3F).

### QTL mapping by pooled-segregant whole-genome sequence analysis.

**FIG 4:**
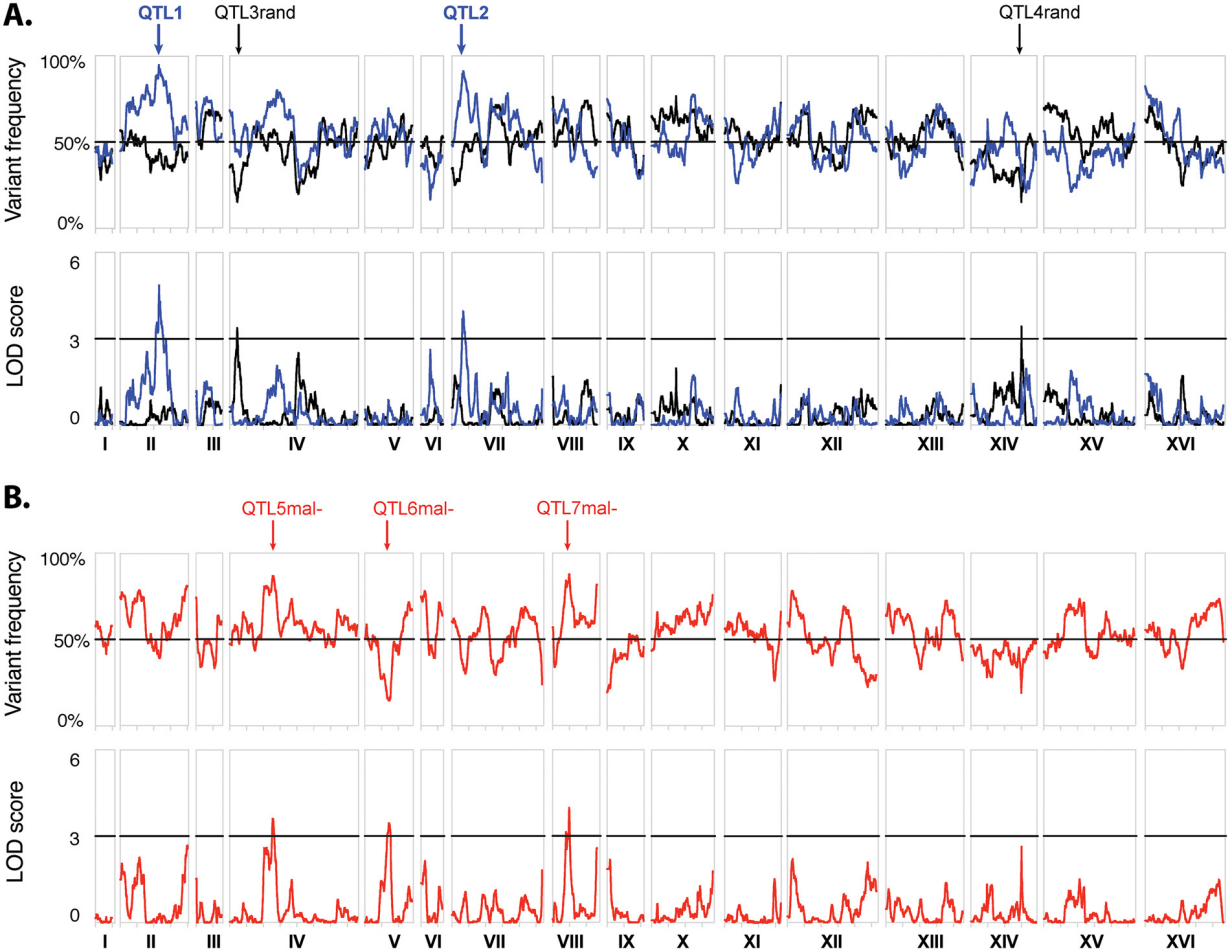
QTL mapping by pooled-segregant whole-genome sequence analysis for high IAAc/Alc ratio in CO_2_ pressurized fermentations. QTL mapping of (A) high IAAc/Alc ratio in CO_2_ pressurized fermentations and (B) lack of maltose fermentation. The regression line of the selected pools is shown in blue and that of the unselected maltose positive pool is shown in black. SNP variant frequency as percentage of that of the superior Seg.63 parent is shown in the upper panel while log_10_ linkage likelihood (LOD score) is shown in the lower panel, as a function of the S. cerevisiae S288c genome position. A threshold LOD score value of 3 has been used as cut-off for significance of the QTLs.

Genomic DNA was isolated from the pool of 28 maltose positive segregants with superior IAAc/Alc ratio and from 2 reference pools each composed of 33 segregants randomly selected with respect to the IAAc/Alc ratio, and either capable or incapable of complete maltose fermentation within 6 days in malt extract wort. The 33 segregants in the 2 reference pools showed a large variability in the IAAc/Alc ratio (42.1-163.5% of the Seg. 63 x ER7A diploid parent). The genomic DNA was subjected to Illumina whole-genome sequencing (BGI). We performed QTL mapping by genome assembly of the sequence reads blasted against the S. cerevisiae S288c reference genome and plotting SNP variant frequency against SNP genomic position to create maps showing linkage disequilibrium along the genome. For the first pool of 28 maltose positive segregants selected for superior IAAc/Alc ratio, 2 QTLs were observed strongly linked to high IAAc/Alc ratio in CO_2_ pressurized fermentations. QTL1 and QTL2 showed a 1-LOD drop-off interval between 457.2 to 479.7 kb (22.5 kb) in chromosome II and 121.4 to 160.4 kp (39.0 kb) in chromosome VII, respectively (Fig. 4A, blue line). Both QTLs were linked to the genome of the superior parent. Neither *ATF1* nor *ATF2* were part of or close to any of the 2 identified QTLs, indicating that the genetic cause for the high IAAc/Alc ratio in the superior parent is to be found outside the production enzymes itself. In contrast, in the pool of 33 maltose positive segregants randomly selected for the IAAc/Alc ratio, QTL1 and QTL2 were absent. Instead, 2 minor QTLs were observed in different positions, QTL1rand in chromosome IV between 75.3 and 101.0 kb and QTL2rand in chromosome XIV between 597.3 and 602.4 kb, linked to the inferior parent (Fig. 4A, black line). QTL1rand and QTL2rand contained no obvious candidate genes related to maltose fermentation, mating capacity or sporulation capacity. Hence, it is unclear why these genomic DNA regions were inadvertently selected in the random pool. The absence of any overlap between the QTLs mapped with the pool of segregants selected for high IAAc/Alc ratio and the pool of randomly selected segregants suggests that there is no link between the IAAc/Alc ratio and maltose fermentation capacity.

To further rule out any bias caused by the preselection of maltose positive segregants, we performed QTL mapping of deficient maltose fermentation capacity using a maltose negative pool, and therefore unselected for the IAAc/Alc ratio (Fig. 4B, red line). In this case, 3 QTLs were observed, QTL1mal- in chromosome IV between 500.5 to 537.4 kb, QTL2mal- in chromosome V between 260.3 to 310.6 kb, and QTL3mal- in chromosome VIII between 189.8 to 205.2 kb (Fig. 4B, red line). All three QTLs observed were also different from the 2 major QTLs mapped for high IAAc/Alc ratio with the maltose positive segregants. Hence, positive and negative maltose fermentation capacity were never linked to high IAAc/Alc ratio. This confirmed that the genetic basis of the 2 traits is unrelated.

### Identification of *MDS3* as the causative gene in QTL2.

**FIG 5:**
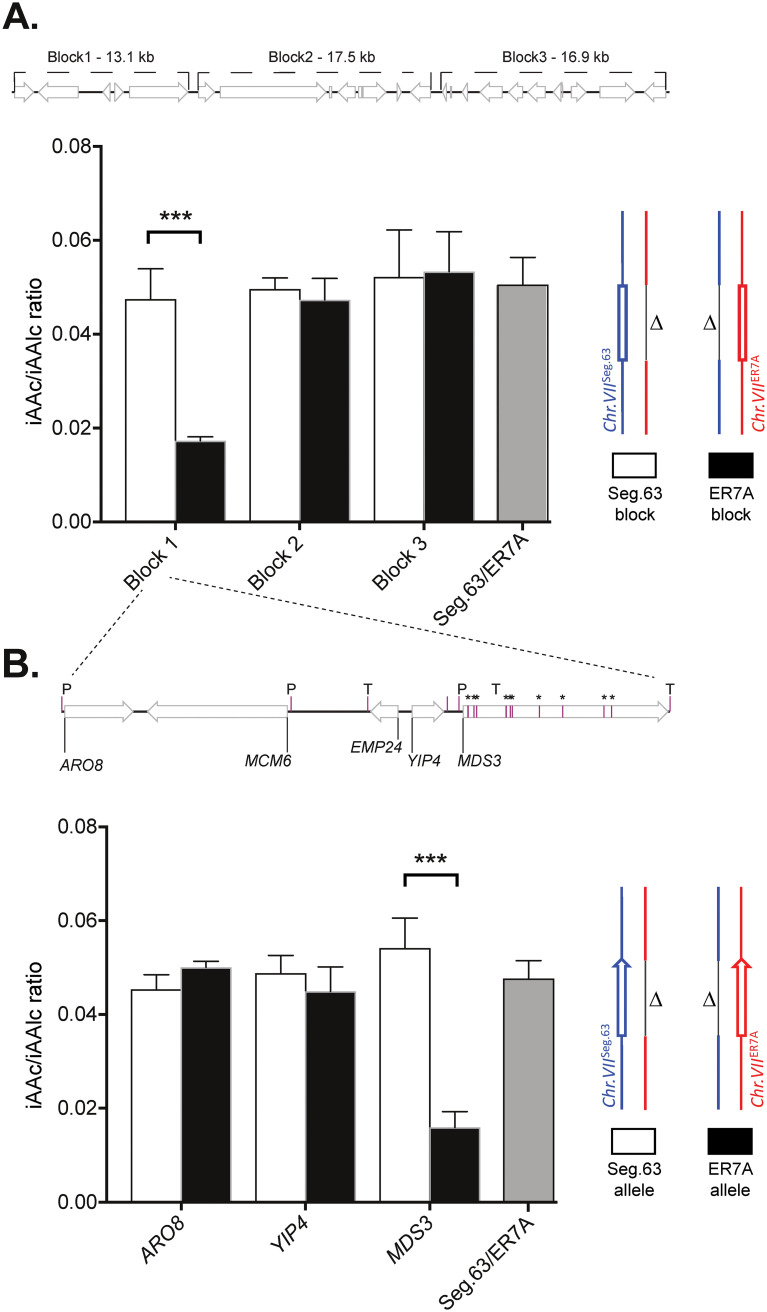
Identification of *MDS3* as the major causative gene in QTL2. QTL1 and QTL2 were the most strongly linked QTLs identified for superior IAAc/Alc ratio in fermentations under CO_2_ pressure. QTL1 was narrow, whereas QTL2 was broader, with the most-linked region (1-LOD interval) being 22.5 and 39 kb, respectively. To narrow down further the area in QTL2, we performed bulk RHA analysis with three blocks of genes. (A) Bulk RHA results for QTL2, identifying block 1 as causative. The position of the blocks is indicated on top. (B) To identify the causative genetic element in block 1 of QTL2, individual gene RHA was performed. Missense (single asterisk), and all combined promoter (P) and terminator (T) mutations are indicated for the genes in block 1. The significance indicated above the bars was determined with a Student's *t* test with correction for multiple testing with false discovery rate of 1%. *, *P* < 0.05; **, *P* < 0.01; ***, *P* < 0.001. The fermentations were carried out in quadruplicate with duplicate fermentations for each of two independent isolates.

To further narrow down the linked area in QTL2, we performed bulk reciprocal hemizygosity analysis (RHA) (36). Five kilobase pairs flanking each side of the predicted 1-LOD drop-off interval were included as a safeguard, resulting in a section of 116,059 to 165,091 bp in chromosome VII for RHA analysis. We divided this region into 3 blocks of 13.1, 17.5 and 16.9 kb, of which the regions from superior haploid Seg.63 or inferior haploid ER7A, respectively, were deleted separately in the Seg.63/ER7A hybrid. The IAAc/Alc ratio was evaluated in CO_2_ pressurized fermentations with the reciprocally deleted hybrid strains. A significant drop in the IAAc/Alc ratio was observed for the strain with deletion of the block 1 fragment (116,059 to 129,161 bp) originating from the superior haploid strain, Seg.63 (Fig. 5A). We next performed single gene RHA for all genes in block 1 and identified the *MDS3* gene as the major causative element in QTL2 (Fig. 5B). The *MDS3* gene contained 10 missense mutations between the Seg.63 and ER7A alleles. The effect of deletion of the *MDS3*^Seg.63^ allele was striking, causing a drop with 71% of the IAAc/Alc ratio (Student's *t* test, *P* = 0.00008). This identified the *MDS3* allele from Seg.63 as the major genetic element responsible for the difference between Seg.63 and ER7A in tolerance of the IAAc/Alc ratio to high CO_2_ pressure. However, the deletion of the *MDS3*^Seg.63^ allele in the hybrid diploid strain also caused a growth defect resulting in slower fermentations that finished after only 6 days in comparison to 4 days for WT strains. This prompted us to investigate the *MDS3* gene further in allele exchange experiments.

### *MDS3* allele replacement in the parental strains Seg.63, ER7A, and Seg.63/ER7A.

**FIG 6:**
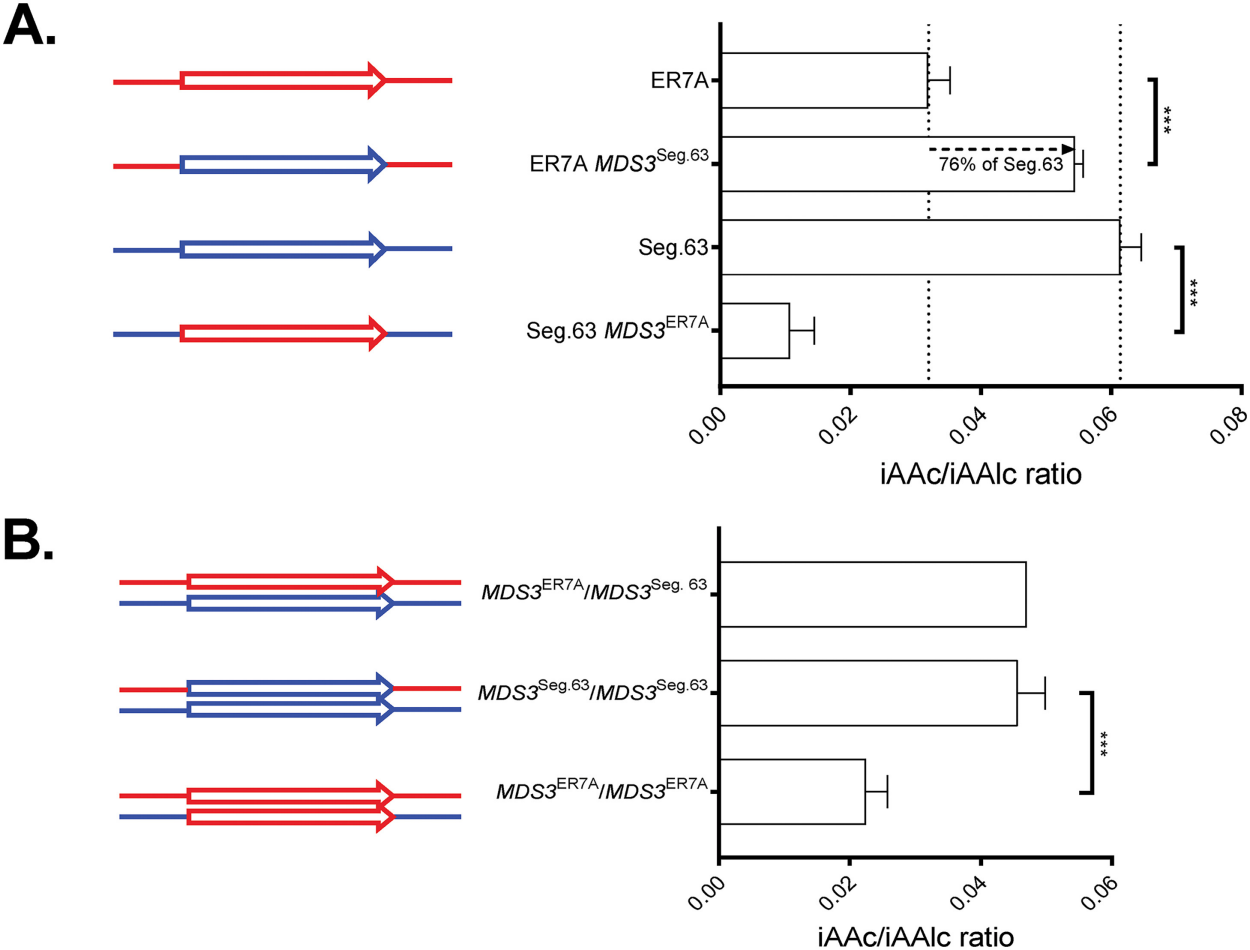
Allele replacement of the *MDS3* gene in the parental strains. The causative effect of the *MDS3* alleles isolated from the superior Seg.63 and inferior ER7A strain was confirmed by allele replacement using CRISPR/Cas9 technology. IAAc/Alc ratio in strains obtained by allele replacement in the Seg.63 and ER7A haploid parent strains (A) and in the Seg.63/ER7A hybrid diploid strain (B). Significance indicated above the bars was determined with a Student's *t* test with correction for multiple testing with false discovery rate of 1%. *, *P* < 0.05; **, *P* < 0.01; ***, *P* < 0.001. The fermentations were carried out in triplicate.

To assess the importance of the *MDS3* gene further, we deleted *MDS3* in Seg.63, ER7A, and the Seg.63/ER7A hybrid with a NatMX selection marker flanked upstream and downstream by 2 Caenorhabditis elegans *lir-2* (G2) protospacer sequences (37) and performed allele exchange through Cas9-mediated cutting at the G2 sites while supplementing with PCR-amplified donor DNA. The causative character of *MDS3* for conferring a superior IAAc/Alc ratio was confirmed by allele replacement in the inferior parent strain ER7A with the superior *MDS3*^Seg.63^ allele, which recovered 76% of the Seg.63 parental phenotype, corresponding to an increase with 71% of the IAAc/Alc ratio (Fig. 6A). Replacement of *MDS3*^ER7A^ in the Seg.63/ER7A hybrid diploid, to provide homozygosity with two *MDS3*^Seg.63/Seg.63^ alleles, did not increase the IAAc/Alc ratio further compared to the unmodified Seg.63/ER7A *MDS3*^Seg.63/ER7A^ strain (Fig. 6B). This supports that the superior *MDS3*^Seg.63^ allele is dominant, at least in the hybrid background. On the other hand, the IAAc/Alc ratio was strongly reduced in the haploid Seg.63 *MDS3*^ER7A^ and hybrid diploid Seg.63/ER7A *MDS3*^ER7A/ER7A^ strains (Fig. 6A and B). The Seg.63 and Seg.63/ER7A strains that only contained 1 or 2 *MDS3*^ER7A^ alleles, respectively, showed a growth defect resulting in slower fermentations that were finished only after 6 days in comparison to the 4 days for the wild-type parental strains. There were no apparent effects of the *MDS3*^Seg.63^ allele on growth or fermentation capacity.

### Engineering of the superior *MDS3*^Seg.63^ allele in a tetraploid lager strain.

**FIG 7:**
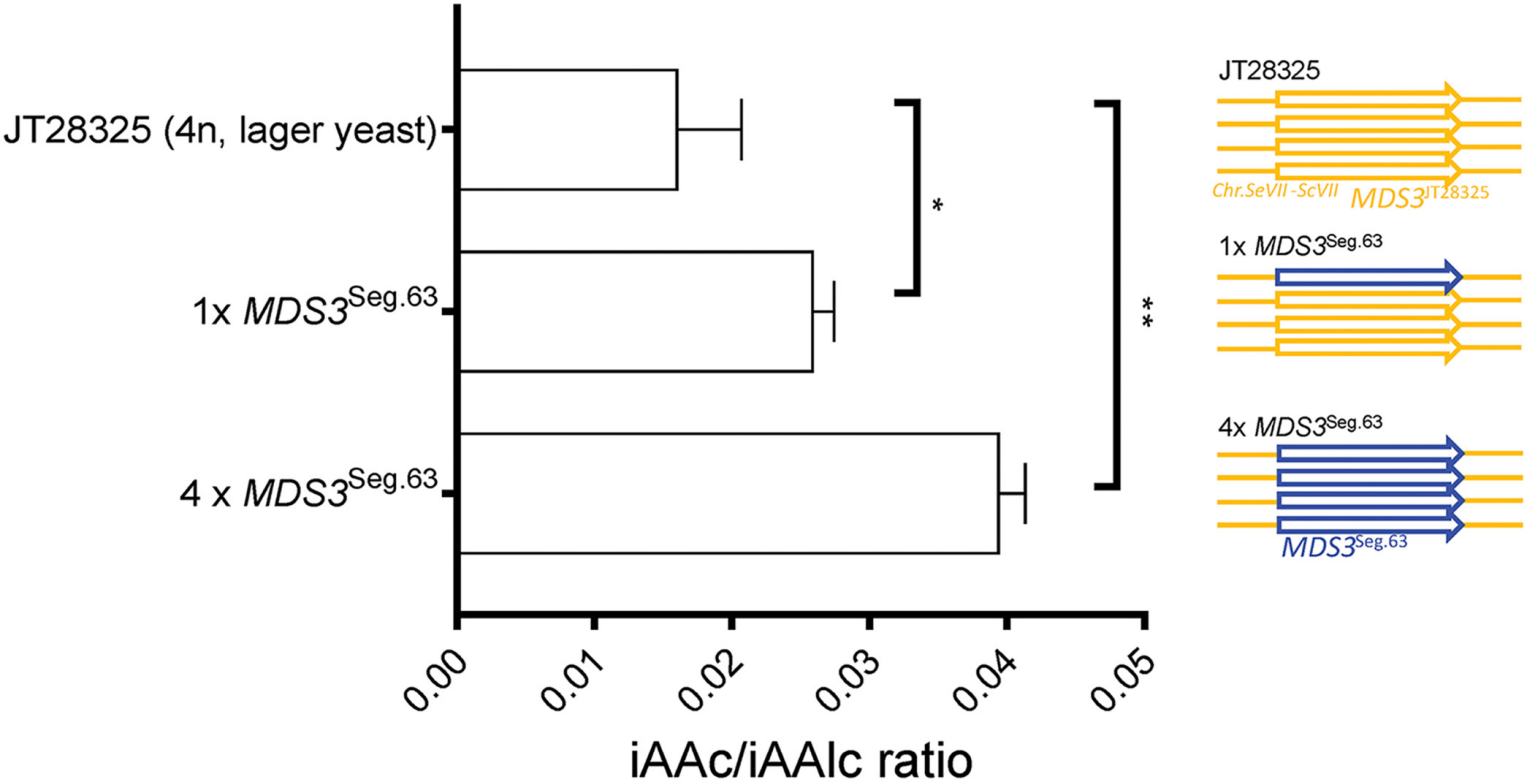
Engineering of the *MDS3*^Seg.63^ allele into the tetraploid lager yeast JT28325. Allele replacement was performed with one or with all 4 alleles in a Frohberg type lager yeast. Significance indicated above the bars was determined with a Student's *t* test with correction for multiple testing with false discovery rate of 1%. *, *P* < 0.05 and **, *P* < 0.01. The fermentations were carried out in triplicate with three independent transformants.

Given the industrial potential of the superior *MDS3*^Seg.63^ allele, we further investigated its applicability to engineer an S. pastorianus lager yeast, JT28325, using CRISPR/Cas9. The Frohberg type S. pastorianus lager yeast originates from a hybridization event between S. cerevisiae and S. eubayanus. It has an approximately tetraploid (aneuploid) genome (38, 39). At the *MDS3* locus, the Frohberg type strain contains 4 copies of the same allele, without any variants in the promoter, open reading frame or terminator region. It has 98.7% sequence similarity to *MDS3* of the Seg.63 and ER7A S. cerevisiae strains. We used the CRISPR/Cas9 protocol described in Materials and Methods, which allowed replacement of all four *MDS3* alleles with the *MDS3*^Seg.63^ allele. Replacement of a single copy out of the 4 identical *MDS3* copies in lager yeast strain JT28325 was performed by first knocking out a single gene and replacing the ORF using the G2 protospacer sequence, as described in Materials and Methods. Both single and quadruple *MDS3* replacement in the tetraploid lager yeast increased the IAAc/Alc ratio significantly, with a higher increase in case of replacement of all 4 alleles (61% and 145% increase, respectively) (Fig. 7). This confirms that the *MDS3*^Seg.63^ allelic variant is dominant, and shows that it is also highly effective for improving the IAAc/Alc ratio in a completely unrelated and industrially highly relevant genetic background.

### Identification of the causative allelic variant, *MDS3*^T2171C^ (Mds3^F724S^).

**FIG 8:**
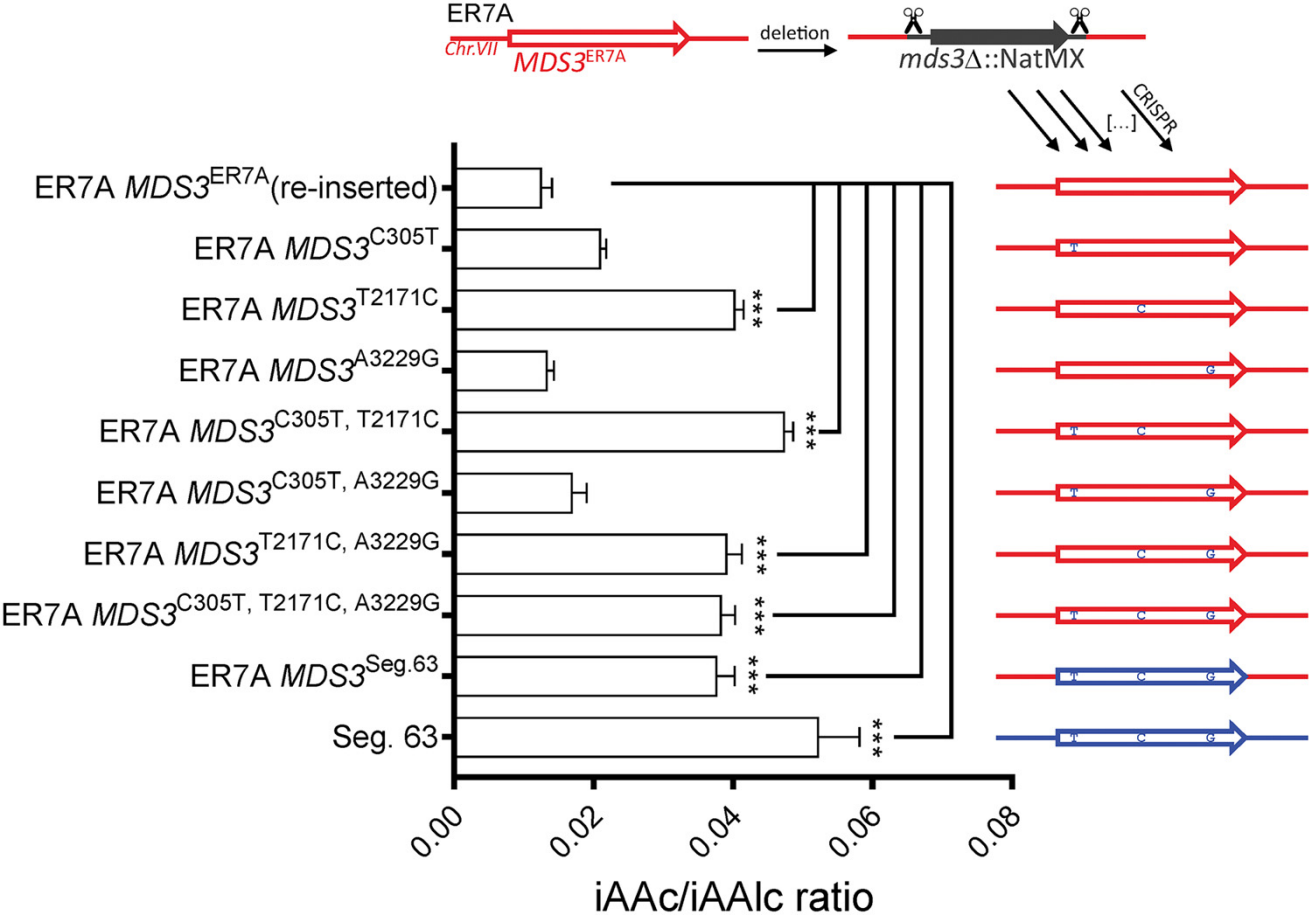
Identification of F724S (T2171C) as the causative SNP variant in *MDS3*^Seg.63^. To identify the causative SNP(s) in the *MDS3* gene, we reintroduced mutant alleles assembled from DNA fragments of *MDS3* containing all possible combinations of SNPs. Significance of the difference with the ER7A reference strain indicated above the bars was determined with a One-way ANOVA test with Dunett’s correction for multiple testing. *, *P* < 0.05; **, *P* < 0.01; ***, *P* < 0.001. The fermentations were carried out in triplicate.

We compared the 10 *MDS3* SNPs between Seg.63 and ER7A in the open reading frame with the sequence of the *MDS3*^JT28325^ allele present in the lager yeast JT28325. This revealed 3 unique missense mutations, C305T (T102M), T2171C (F724S), and A3229G (I1077V) in the superior *MDS3*^Seg.63^ allele (Table 1) compared to the *MDS3* alleles in the JT28325 strain. To identify which (combination) of the SNPs were causative, we performed genome editing of the *MDS3* loci in the inferior ER7A strain using CRISPR/Cas9 and linear donor DNA constructs, containing all possible SNP combinations. Strikingly, the only causative SNP variant was T2171C, causing an amino acid change of phenylalanine to serine at position 724 in the Mds3 gene product. Any combination that included the T2171C variant yielded an IAAc/Alc ratio indistinguishable from that obtained after exchange of the entire *MDS3* allele from Seg.63 (Fig. 8). Except for a slight increase in the ethyl octanoate level (“apple” descriptor), introduction of the T2171C variant did not affect the production of any other flavor compounds (Table S1). Hence, we have identified a single SNP, T2171C, in the *MDS3* gene, which can generate by itself a highly significant and specific enhancement of the IAAc/Alc ratio in fermentations under high CO_2_ pressure.

**TABLE 1:** Overview of all missense SNPs in the *MDS3* gene and the corresponding amino acid changes in Mds3^*a*^

Position in ORF	Amino acid change (ER7A > seg.63)	ER7A	SEG63	JT28325
117	E39D	A	C	C
244	T82A	A	G	G
305	T102M	C	T	C
943	K315E	A	G	G
1033	S345P	T	C	C
1075	H359Y	C	T	T
1664	F555S	T	C	C
2171	F724S	T	C	T
3064	T1022A	A	G	G
3229	I1077V	A	G	A

a The nucleotide position in the ORF, corresponding amino acid change, and nucleotide variant at that position are indicated for the inferior ER7A, superior Seg.63, and the JT28325 lager yeast. Shading indicates the three unique missense mutations in *MDS3*.

## DISCUSSION

The CO_2_ inhibition of flavor production in large cylindroconical fermentors has long been recognized as one of the most important challenges at industrial scale in modern brewing. Nevertheless, yeast strains maintaining appropriate flavor production under high CO_2_ pressure have not yet been developed for industrial brewing conditions. Very little is also known about the molecular basis and inter-strain variability in tolerance of flavor production to high CO_2_ pressure. The main outcome of the present study is the identification of a single SNP variant in the *MDS3* gene, *MDS3*^T2171C^ (encoding Mds3^F724S^), that dominantly confers higher tolerance to CO_2_-induced reduction of the isoamyl acetate production yield. CRISPR/Cas9 mediated engineering of the novel *MDS3* allele into a tetraploid lager yeast by allele exchange enhanced the IAAc/Alc ratio by 145% in a beer fermentation under elevated CO_2_ pressure. Generally, for lager beer, a high IAAc/Alc ratio is desired because it expresses more the refreshing fruity banana type of aroma instead of the alcoholic aroma from higher alcohols. When application of genome editing technology on brewing strains is unfavorable, a brewing yeast with the superior characteristic could also be obtained by marker-assisted breeding.

The high hydrostatic pressure in large industrial cylindroconical fermentors leads to a physical agitation effect by the CO_2_ bubbles accumulating at high depths, which inhibits the formation of esters (1, 8), and exerts a physical pressure on the yeast cell wall, which is also likely to affect the metabolism of the cells. Further fermentation tests under high hydrostatic and CO_2_ pressure, with agitation and at lower temperature (affecting membrane fluidity) would therefore reveal the general potential of the novel *MDS3* variant to provide its beneficial effect in different industrial fermentation conditions. Surprisingly, we observed that CO_2_ overpressure above 0.65 bar caused no apparent inhibitory effect on the IAAc/Alc ratio (Fig. 2D), which may illustrate the limitations of the experimental setup and a larger variability of flavor production at higher pressures. A CO_2_ overpressure of 1 bar was estimated to correspond to approximately 10 m depth in the fermentor for pilot scale fermentations (11). We may therefore have investigated pressures that are below what the yeast experiences at least at the bottom of the largest industrial fermenters. It is, thus, possible that higher concentrations of CO_2_ would have revealed additional new genes. Using our laboratory scale fermentation system, we observed a reproducible, strong and strain-dependent inhibition of the AATase activity (as reflected in the IAAc/Alc ratio) at 0.65 bar CO_2_ pressure, with minimal inhibition on fermentation performance, and fermentation under higher CO_2_ pressures was therefore not investigated further in this study.

We have demonstrated that the superior variant of *MDS3*, *MDS3*^T2171C^, specifically increases the isoamyl and isobutyl acetate levels and does not affect the production of other aroma compounds (Table S1). These acetate esters are uniquely formed through esterification of the fusel alcohol precursors isoamyl and isobutyl alcohol by the AATase enzymes Atf1 and Atf2 using acetyl-CoA as a co-substrate (40). Whereas isoamyl and isobutyl alcohol levels were found to be unchanged, it is possible that acetyl-CoA might have been increased through introduction of the *MDS3*^T2171C^ variant. However, this appears unlikely since none of the other aroma compounds showed altered levels. Moreover, previous research has revealed that the major limiting factor for isoamyl acetate productivity is the low expression level of the AATase genes *ATF1* and *ATF2* (13). We therefore suggest that the *MDS3*^T2171C^ (Mds3^F724S^) variant might act by elevating the expression level of the AATase genes. The superior haploid Seg.63 segregant and the hybrid Seg.63/ER7A diploid showed a growth and fermentation defect when they were downgraded with the *MDS3*^ER7A^ allele. This indicates that the *MDS3* gene plays a vital role in at least one important cellular pathway, different from those involved in regulation of isoamyl acetate production, and that this effect is dependent on the genetic background of the strain. Mds3 appears to be a positive regulator of the Target Of Rapamycin (TOR) pathway, since an *mds3* knockout S. cerevisiae strain is highly sensitive to rapamycin, a TORC1 inhibitor (41). In Candida albicans, Mds3 interacts with the downstream TORC1 effector and PP2A-related protein phosphatase Sit4, linking the Mds3 protein to nutrient sensing through TORC1 (41). High TOR activity is correlated with strong growth, partly due to high expression of ribosomal genes involved in translation (20). The major AATase *ATF1* gene is strongly regulated by the Rap1 activator/repressor transcription factor, and disruption of the downstream TORC1 effector, Sch9, reduces its expression level (16). Interestingly, the same F724S mutation was found in a heterozygous Mds3^WT^/Mds3^F724S^ diploid laboratory yeast strain after evolutionary adaptation to a low glucose level in the medium for 264 generations (42). In this screen, the Mds3^F724S^ mutation was identified together with mutations in nine other genomic loci, including *TOR1* (Tor1^T2231K^), but no molecular link with Tor1 or any of the other gene products was established. The Mds3^F724S^ mutation was only present in 1 out of 5 clones of which the genome had been completely sequenced. The remainder of the clones contained mutations in other loci, indicating convergent evolution of the phenotype by modulation of other pathways, such as the cAMP/Ras and TOR pathways. Nevertheless, all clones isolated from the evolved cultures showed increased fitness with low glucose and acetate concentrations, but on the other hand lower performance under nitrogen-limited conditions, indicating a trade-off for the competitive advantage under carbon limitation (42). This is consistent with identification of an *MDS3* allele yielding an improved fermentation rate with poor nitrogen sources, linking nitrogen signaling and glycolytic flux (43). We have performed experiments in low nitrogen wort using strains modified with the *MDS3*^T2171C^ allele, but did not find any effects on growth and fermentation performance in brewing conditions, nor was the positive effect of *MDS3*^T2171C^ reduced in low nitrogen conditions (data not shown). This may suggest that the trade-off for improved growth with low glucose and the poor performance with low nitrogen is a polygenic trait that requires genetic modifications in other loci than *MDS3* alone.

The C. albicans *MDS3* gene has been linked to nutrient and pH dependent pseudohyphae formation and has been reported as being essential for growth in alkaline minimal medium (together with its paralog *PMD1*), suggesting a role of *MDS3* in pH regulation (41, 44). The growth inhibitory effects of CO_2_ are to some extent caused by direct inhibition of metabolic enzymes, lowering of the intracellular pH and impairing mitochondrial function once the CO_2_ (and bicarbonate) is inside the cells (10, 22–24). It is tempting to speculate that the Mds3^F724S^ allele provides tolerance to lower intracellular pH and sustains high TOR activity in the presence of high levels of dissolved CO_2_.

**FIG 9:**
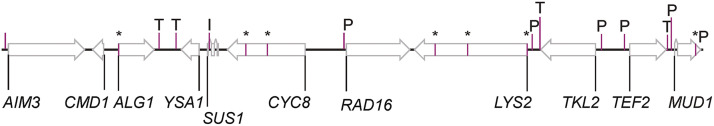
SNP variants in QTL1. The most strongly linked area in QTL1 with the different genes and their mutations is indicated. Missense (single asterisk), and all combined promoter (P), terminator (T), and intron (I) mutations are indicated.

In addition to the discovery of the superior *MDS3* variant, we have identified a second QTL, QTL1, in chromosome II (Fig. 4), which was not investigated further due to the strong effect of *MDS3*. This 22.5 kb genomic region contained 10 genes with mutations in the promoter, terminator or missense mutations in the ORFs (Fig. 9). The best candidate appears to be *CYC8* (previously annotated as *SSN6*), which contains two missense mutations in the ORF, H467Q and Q733R. Based on the publicly available genome sequences in GenBank, the H467Q variant is a common variant found in many yeast strains, whereas the latter is present uniquely in four sake yeast strains. Cyc8 is a co-repressor which forms a complex with Tup1 downregulating *ATF1* expression through the oxygen responsive transcription factor, Rox1 (17). However, molecular characterization of this locus is necessary to confirm the causality.

### Conclusions.

Our work has revealed the strong potential of polygenic analysis to identify unique alleles, like *MDS3^T2171C^*, that confer tolerance to inhibition of yeast flavor production by high carbon dioxide pressure. It also showed that through CRISPR/Cas9 mediated allele exchange these superior alleles can be used successfully to enhance tolerance of yeast flavor production to high carbon dioxide pressure in commercial lager brewing strains, creating cisgenic superior industrial strains that are apparently not significantly affected in other traits important for brewing. The knowledge on the superior alleles can also be used in strain selection and marker-assisted strain breeding programs to develop commercial yeast strains displaying high tolerance to carbon dioxide inhibition of flavor production. Our work again illustrates the strong potential of the wide biodiversity present in the large S. cerevisiae strain collections to provide unique alleles for targeted strain improvement.

## MATERIALS AND METHODS

### Strains and plasmids.

Yeast strains and plasmids used in this work are shown in Table 2.

**TABLE 2:** Yeast strains and plasmids used in this study

Yeast strain	Description	Source and/or reference
Kyokai no. 1	Industrial saké yeast; MAT**a**/**α**	National Research Institute of Brewing, Japan
ER7A	Haploid segregant of Ethanol Red (industrial bioethanol strain), used for QTL analysis; MAT**a**	MCB, KU Leuven (54)
Seg.63	Haploid segregant of Kyokai no. 1, used for QTL analysis; MAT**α**	This study
ER7A/Seg.63	Hybrid diploid strain obtained by crossing ER7A and Seg.63	This study
JT28332	Ale yeast, S. cerevisiae	MCB, KU Leuven
JT28333	Lager yeast; tetraploid S. pastorianus, Frohberg	MCB, KU Leuven
JT28334	Lager yeast; S. pastorianus, Frohberg	MCB, KU Leuven
JT28335	Lager yeast; S. pastorianus, Frohberg	MCB, KU Leuven
JT28325	Lager yeast; S. pastorianus, Frohberg	MCB, KU Leuven
JT28325 1 x *MDS3*^Seg.63^	JT28325 with one out of four *MDS3* alleles replaced with *MDS3*^Seg.63^	This study
JT28325 4 x *MDS3*^Seg.63^	JT28325 with all four *MDS3* alleles replaced with *MDS3*^Seg.63^	This study
ER7A *MDS3*^Seg.63^	Haploid ER7A, *MDS3* allele replaced with *MDS3*^Seg.63^	This study
Seg.63 *MDS3*^ER7A^	Haploid Seg.63, *MDS3* allele replaced with *MDS3*^ER7A^	This study
ER7A/Seg.63 *MDS3*Seg.63/*MDS3*Seg.63	Hybrid diploid ER7A/Seg.63, homozygous for *MDS3*^Seg.63^	This study
ER7A/Seg.63 *MDS3*ER7A/*MDS3*ER7A	Hybrid diploid ER7A/Seg.63, homozygous for the *MDS3*^ER7A^	This study
ER7A/Seg.63 QTL2 block1^Seg.63^	RHA strain ER7A/Seg.63, ER7A block 1 in QTL2 (chrVII: 116,059 to 129,161 bp) replaced with KanMX cassette	This study
ER7A/Seg.63 QTL2 block1^ER7A^	RHA strain ER7A/Seg.63, Seg.63 block 1 in QTL2 (chrVII: 129,884 to 147,394 bp) replaced with KanMX cassette	This study
ER7A/Seg.63 QTL2 block2^Seg.63^	RHA strain ER7A/Seg.63, ER7A block 2 in QTL2 (chrVII: 129,884 to 147,394 bp) replaced with KanMX cassette	This study
ER7A/Seg.63 QTL2 block2^ER7A^	RHA strain ER7A/Seg.63, Seg.63 block 2 in QTL2 (chrVII: 129,884 to 161,219 bp) replaced with KanMX cassette	This study
ER7A/Seg.63 QTL2 block3^Seg.63^	RHA strain ER7A/Seg.63, ER7A block 3 in QTL2 (chrVII: 148,232 to 165,084 bp) replaced with KanMX cassette	This study
ER7A/Seg.63 QTL2 block3^ER7A^	RHA strain ER7A/Seg.63, Seg.63 block 3 in QTL2 (chrVII: 148,232 to 165,084 bp) replaced with KanMX cassette	This study
ER7A/Seg.63 *ARO8*^Seg.63^	RHA strain ER7A/Seg.63, *ARO8*^ER7A^ allele replaced with KanMX cassette	This study
ER7A/Seg.63 *ARO8*^ER7A^	RHA strain ER7A/Seg.63, *ARO8*^Seg.63^ allele replaced with KanMX cassette	This study
ER7A/Seg.63 *YIP4*^Seg.63^	RHA strain ER7A/Seg.63, *YIP4*^ER7A^ allele replaced with KanMX cassette	This study
ER7A/Seg.63 *YIP4*^ER7A^	RHA strain ER7A/Seg.63, *YIP4*^Seg.63^ allele replaced with KanMX cassette	This study
ER7A/Seg.63 *MDS3*^Seg.63^	Hybrid diploid RHA strain ER7A/Seg.63, *MDS3*^ER7A^ allele replaced with KanMX cassette	This study
ER7A/Seg.63 *MDS3*^ER7A^	Hybrid diploid RHA strain ER7A/Seg.63, *MDS3*^Seg.63^ allele replaced with KanMX cassette	This study
ER7A *mds3*Δ::*NatMX*	Haploid ER7A, MDS3 allele knock-out with NatMX cassette flanked with G2 gRNA target sites	This study
ER7A *MDS3*^WT^ (re-inserted)	Haploid ER7A, *MDS3*^ER7A^ re-inserted into the *MDS3* locus	This study
ER7A *MDS3*^C305T^	Haploid ER7A, Gibson assembled *MDS3*^C305T^ inserted into the *MDS3* locus	This study
ER7A *MDS3*^T2171C^	Haploid ER7A, Gibson assembled *MDS3*^T2171C^ inserted into the *MDS3* locus	This study
ER7A *MDS3*^A3229G^	Haploid ER7A, Gibson assembled *MDS3*^A3229G^ inserted into the *MDS3* locus	This study
ER7A *MDS3*^C305T, T2171C^	Haploid ER7A, Gibson assembled *MDS3*^C305T, T2171C^ inserted into the *MDS3* locus	This study
ER7A *MDS3*^C305T, A3229G^	Haploid ER7A, Gibson assembled *MDS3*^C305T, A3229G^ inserted into the MDS3 locus	This study
ER7A *MDS3*^T2171C, A3229G^	Haploid ER7A, Gibson assembled *MDS3*^T2171C, A3229G^ inserted into the MDS3 locus	This study
ER7A *MDS3*^C305T, T2171C, A3229G^	Haploid ER7A, Gibson assembled *MDS3*^C305T, T2171C, A3229G^ inserted into the MDS3 locus	This study
Plasmid		
p414-TEF1p-Cas9-CYC1t	Cas9 expression plasmid	48
p426-SNR52p-gRNA. CAN1.Y-SUP4t	Guide RNA expression plasmid	48
pTEF-Cas9-KanMX	p414-TEF1p-Cas9-CYC1t with KanMX selection marker	This study
p426-sgRNA-HphMX	p426-SNR52p-gRNA. CAN1.Y-SUP4t with HphMX selection marker and EcoRV restriction flanking gRNA	This study
p426-sgRNA-NatMX	p426-SNR52p-gRNA. CAN1.Y-SUP4t with NatMX selection marker and EcoRV restriction flanking gRNA	This study
pJET-2xgRNA	pJET bacterial vector with -structural gRNA-SUP4t-SNR52p- for PCR amplification of constructs with two gRNAs	This study
p426-*MDS3*^Seg.63^-donor	Multicopy p426 donor plasmid (HphMX) with *MDS3*^Seg.63^	This study
p426-sgRNA-2x*MDS3*-NatMX	p426-SNR52p-gRNA. CAN1.Y-SUP4t with NatMX selection marker and expression of two gRNAs targeting *MDS3*	This study
p426-sgRNA-G2-HphMX	p426-SNR52p-gRNA. CAN1.Y-SUP4t with HphMX selection marker and expression of G2 gRNAs	This study
p426-sgRNA-G2-NatMX	p426-SNR52p-gRNA. CAN1.Y-SUP4t with NatMX selection marker and expression of G2 gRNAs	This study

### Molecular biology methods.

Yeast cells were transformed by electroporation (45). Standard molecular biology protocols were used in this work.

### Cultivation media.

Yeast cells were grown at 30°C in YPD medium [2% (wt/vol) glucose, 2% (wt/vol) peptone, 1% (wt/vol) yeast extract] with shaking at 200 rpm. For solid nutrient plates, 1.5% (wt/vol) Bacto agar was added. Escherichia coli cells (DH5, Invitrogen) were grown at 37°C in Luria Broth (LB) medium containing 0.5% (wt/vol) yeast extract, 1% (wt/vol) Bacto tryptone, and 1% (wt/vol) sodium chloride (pH 7.5). For solid nutrient plates, 1.5% (wt/vol) Bacto agar was added. Selection of transformants was performed in the presence of 100 mg/L ampicillin.

### Screening in YP250-10%Glu media.

Flavor compound screening was performed in YP250 (0.27% yeast extract, Merck, 0.54% Bacto peptone, Oxoid, to a total predicted nitrogen content of 250 mg/L and adjusted to pH 4.5 with concentrated hydrochloric acid) containing 10% (wt/vol) glucose. The predicted nitrogen content was based on information of titratable nitrogen from the suppliers. The collection of strains was pre-cultured in 1 mL YP250-2%Glu, and fermentations were inoculated by volume with 0.5 mL culture in total volumes of 100 mL (35).

### A laboratory scale fermentation system with high CO_2_ pressure.

The design of the system was done in collaboration with the suppliers of the equipment (Pneuvano, Wommelgem and KU Leuven Glasblazerij). The pressure resistant bottles, stirring rod, tubing with sterilization filter, safety valve and plug were autoclaved as a whole (assembled). After cooling, the safety release valves were set to 1 bar before addition of the medium. The rubber stops were penetrated by 2 glass tubes, one for CO_2_-release and one for sampling, and the tubes were sealed with sterile cotton and a plastic tube with a clamp, respectively.

### Malt extract medium.

Malt extract medium consisting of 166 g/L of malt extract (Brewferm spraymalt 8 EBC) supplemented with 0.5 mg/L ZnSO_4_, was autoclaved at 110°C for 15 min. After autoclaving, the malt extract medium was cold settled overnight and filtered through a nylon filter (GE Healthcare) to remove insoluble precipitates. The final gravity of the malt extract medium was 15°P. Before fermentation, the medium was over-aerated by purging with pure oxygen supplied in a gas bottle to provide enough oxygen for the biosynthesis of unsaturated fatty acids. The oxygen level was approximately 20 mg/L, measured by an HQ30D dissolved oxygen meter with an LDO101 luminescent dissolved oxygen sensor (HACH).

### Inoculation of beer fermentations.

The cells were inoculated from fresh YPD-plates into liquid YPD medium in 3 mL volume (test tubes) and grown for 24h at 30°C with shaking at 200 rpm. 500 μL cell culture was subsequently transferred to 5 mL of malt extract (test tubes) and grown for 24h at 30°C with shaking at 200 rpm. The optical density was measured and 100 mL of malt extract was inoculated to an OD_600_ of 1 in 300 mL shaking flasks. The cultures were grown for 2 days at 30°C with shaking at 200 rpm.

### Assembly of pools, whole-genome sequence analysis, and QTL mapping.

All strains were cultured separately in 4 mL cultures in 6-well plates for 2 days at 30°C with shaking at 200 rpm in YPD containing 100 mg/L ampicillin and 10 mg/L doxycyclin. The parent strains were grown in 32 mL cultures (8 × 4 mL in 6-well plates). The antibiotics were added to avoid any possibility of bacterial contamination. The mixing was done based on optical density (OD_600_). Due to cell clumping the cultures were first sonicated to obtain a reliable value corresponding with the cell number obtained by colony counting. Genomic DNA was extracted and purified with the “Masterpure Yeast DNA purification kit” from Epicentre in order to obtain high quality genomic DNA.

The DNA was measured with the PicoGreen method (Quant-iT kit, Invitrogen). All the samples contained over 15 μg of DNA, which was subsequently sent to BGI (Hong Kong) for Illumina HiSeq2000 sequence analysis.

Assembly and mapping were done with NGSEP (Next Generation Sequencing Eclipse Plugin) (46) and linkage analysis was performed with MULTIPOOL (47). A LOD score above 3 was considered indicative for the presence of a QTL.

### Reciprocal hemizygosity analysis.

The deletion constructs for bulk RHA were amplified from the Euroscarf laboratory strain (BY4741) deletion collection according to the split marker method. This includes amplification and transformation of two PCR products per genomic target, each containing half of a KanMX marker cassette (split) with a 552 bp overlapping region between the two amplicons. The gene blocks were selected according to the availability of deletion strains and were chosen to have a size of 14 to 18 kb. To ensure efficient homologous recombination for the large deletions, we used long 0.4–1 kb flanking regions. For single gene RHA, 50 bp flanking regions were used.

### CRISPR/Cas9 mediated genome editing.

To perform direct allele replacement of the *MDS3* gene in prototrophic yeast strains, we exchanged auxotrophic *URA3* and *TRP1* markers in the Cas9 guide RNA expression plasmids developed by DiCarlo et al. (48) with antibiotic selection markers. For cutting in 2 genetic loci, we constructed a plasmid by PCR amplification of fragments containing the first target guide RNA sequence fused to the trans-activating crRNA, tyrosine tRNA *SUP4* terminator, and a snoRNA *SNR52* promoter fused to the second guide RNA target sequence (gRNA1-*SUP4*t-*SNR52*p-gRNA2). Single gRNA oligo duplexed or 2xgRNA PCR fragments were inserted through Gibson cloning into the guide RNA expression plasmids for expression of 2 guide RNAs under the *SNR52* promoter and *SUP4*/*CYC1* terminators. The backbone p414-TEF1p-Cas9-CYC1t and p426-SNR52p-gRNA. CAN1.Y-SUP4t plasmids were a gift from George Church (Addgene plasmid # 43802 and 43803).

Single allele replacement was done based on gene deletion by homologous recombination with a NatMX antibiotic marker flanked upstream and downstream by two Caenorhabditis elegans *lir-2* (G2, 5′-GGATGAGAATCTGACAAAGG) protospacer sequences (37). Deletion of the *MDS3* ORF in ER7A and Seg.63 was carried with the primers *MDS3*-A1 FWD, TAAGGCAGACTCCGTGGAGTGTAAGAGAAGTTCAAAGCAAGGTTAGGCTTGTGGTCGGCTGGAGATCGG and *MDS3*-A2 REV, GAATTTGAATTGTCCTGAGCTGACCTGGTCTGCCCGCTTCGATAAACTGCAGCCGTTATGGCGGGCATC, containing a 50 bp homology region up- and downstream of the ORF, and A1/A2 adaptors for amplification of the G2-NatMX-G2 cassette. The G2 gRNA targets were then subsequently targeted for efficient Cas9-mediated cutting, which allowed screening for loss of the antibiotic resistance marker, increasing the success rate for insertion of the alternative full-length *MDS3* allele. Direct replacement of the *MDS3* gene was performed using the following guide RNAs: 5′-GGGTAGCAGAAGCAAGCGGA, targeting the first (synonymous) mutation in the ORF; 5′-GATGTATAGCAGCATATTCT, targeting position 63 bp downstream of the ORF in the terminator.

CRISPR/Cas9 genome editing was commenced by first transforming yeast with a low copy number plasmid for stable and constitutive expression of the Cas9 endonuclease. Next, gRNA plasmids specific for the Seg.63 or ER7A *MDS3* alleles or the G2 sequence were co-transformed with a PCR-amplified donor DNA (1 μg). *MDS3* replacement with the marker cassette (targeting G2) was highly efficient in the haploid strains Seg.63 and ER7A (79% and 76%), but much less efficient for single replacement in the diploid Seg.63/ER7A hybrid (9–11%). The replacement efficiency was also very low in an unrelated diploid brewing yeast (2%) and unsuccessful in triploid and tetraploid strains, even after screening of 347 transformants. This is consistent with a previous report showing that CRISPR/Cas9 modification efficiency using the plasmids developed by DiCarlo et al. (49) is lower for diploids than haploids. This is likely at least to some extent due to a strain specific shortfall of guide RNA expression observed in yeasts with higher ploidy (50, 51). On the other hand, the rate of successful replacement observed for the diploid Seg.63/ER7A hybrid was much lower than expected probably due to the remaining *MDS3* allele in the genome acting more efficiently as donor DNA than the PCR amplified marker cassette. Because of the likely competition between PCR amplified donor template and chromosomal DNA for homologous repair, we tested whether transformation of the donor DNA on a 2-micron multi-copy plasmid (p426) could provide higher efficiency of correct repair. We therefore first transformed the lager yeast strain JT28325 with the multi-copy plasmid p426-*MDS3*^Seg.63^-donor, containing the superior *MDS3*^Seg.63^ allele, and subsequently with plasmids expressing the Cas9 endonuclease and 2 guide RNAs targeting the first mutation in the open reading frame and terminator. Transformation of the donor DNA plasmid prior to the guide RNA, without insertion of an antibiotic marker into the genome, increased the efficiency of direct allele exchange from 0% to 42% for replacement of one or more alleles and 8% for replacement of all four *MDS3* alleles in the tetraploid JT28325 lager yeast.

### Construction of variants of the *MDS3*^Seg.63^ allele.

To identify the causative variant in *MDS3*, we made linear donor DNA constructs with the high-fidelity NEB assembly mix, based on manufacturers instructions. We created four blocks covering the entire *MDS3* ORF and flanked by 200 and 81 bp up- and downstream of the *mds3*::G2-NatMX-G2 cassette. This was done by PCR amplification of fragments containing variants with the following primers: Block1 FWD, 5′-AAAGTCTATTTCAAGTTCACAG; Block1 REV (*MDS3*^305C^), 5′-TCTAGACATCAAGTCTAAGAAAAAC**G**TCTC; Block1 REV (*MDS3*^305T^), 5′-TCTAGACATCAAGTCTAAGAAAAAC**A**TCTC; Block2 FWD: 5′-GTTTTTCTTAGACTTGATGTCTAGA; Block2 REV (*MDS3*^2171T^), 5′-CGCTTTTTCCTTGAAAGGTACTCTG**A**AAA; Block2 REV (*MDS3*^2171C^), 5′-CGCTTTTTCCTTGAAAGGTACTCTG**G**AAA; Block3 FWD, 5′-CAGAGTACCTTTCAAGGAAAAAGCG; Block3 REV (*MDS3*^3229A^), 5′-AAGGTCTCCATCAACGAAGTACATA**T**TAAG; Block3 REV (*MDS3*^3229G^), 5′-AAGGTCTCCATCAACGAAGTACATA**C**TAAG; Block4 FWD, 5′-TATGTACTTCGTTGATGGAGACCTT; Block4 REV: 5′-GGACGTAGCGGTCTATGG. Variants contained in primers are indicated in bold. The Gibson overlap was 25 bp with at least 50°C annealing temperature. After fusion of the fragments by Gibson assembly for 1 h at 50°C, the products were purified to remove primer DNA and re-amplified with Block1 FWD and Block4 REV primers to create sufficient DNA (~5 μg) for transformation into the ER7A haploid yeast.

### Headspace GC-FID analysis.

Headspace gas chromatography coupled with flame ionization detection (GC-FID) was used to measure flavor compounds at the end of the fermentation. Samples were collected and centrifuged at 3500 rpm for 5 min. Then, 5 mL of the supernatant was collected into 25-mL vials and analyzed using a gas chromatograph with a headspace sampler (Triplus RSH, Thermo Scientific). The headspace was equilibrated by shaking and incubation for 10 min at 60°C and then injected into a polyethylene glycol column (Restek Stabilwax, 60 m x 0.25 mm x 0.25 μm).

Injection block and flame ionization detector temperatures were kept constant at 220 and 250°C, respectively. Oven temperature was kept at 40°C for 2 min, then increased to 240°C at a rate of 15°C/min. Helium was used as carrier gas at a flow rate of 2.0 mL/s. GC operating conditions were used as follows: injection volume 1 mL; split rate 1:25; split flow 50 mL/min.

### Mating type determination, sporulation, and tetrad dissection.

Standard procedures were used for sporulation and tetrad dissection (52) and for mating type determination by PCR with primers for MAT**a** and MAT**α** DNA at the MAT locus (53).

### Data availability.

All sequence data have been submitted to the NCBI Sequence Read Archive (SRA) (http://trace.ncbi.nlm.nih.gov/Traces/sra/sra.cgi) with the identifier SRP148958 under Bioproject PRJNA473077.

## References

[1] Meilgaard MC. 2001. Effects on flavour of innovations in brewery equipment and processing: a review. J Inst Brew 107:271–286. 10.1002/j.2050-0416.2001.tb00098.x.

[2] Masschelein CA. 1994. State-of-the-art and future developments in fermentation. J the American Society of Brewing Chemists 52:28–35. 10.1094/ASBCJ-52-0028.

[3] Knatchbull FB, Slaughter JC. 1987. The effect of low CO_2_ pressures on the absorption of amino acids and production of flavour-active volatiles by yeast. J the Inst Brewing 93:420–424. 10.1002/j.2050-0416.1987.tb04530.x.

[4] Renger RS, Hateren S, Luyben KCAM. 1992. The formation of esters and higher alcohols during brewery fermentation; the fffect of carbon dioxide pressure. J Inst Brew 98:509–513. 10.1002/j.2050-0416.1992.tb01137.x.

[5] Kruger L, Pickerell ATW, Axcell B. 1992. The sensitivity of different brewing yeast strains to carbon dioxide inhibition: fermentation and production of flavour-active volatile compounds. J Inst Brewing 98:133–138. 10.1002/j.2050-0416.1992.tb01100.x.

[6] Ulenberg GH, Gerritson H, Huisman J. 1972. Experiences with a giant cylindrico-conical tank. Master Brewers Ass Amer Tech Quart 117–122.

[7] Rice JF, Chicoye E, Helbert JR, Garver J. 1977. Inhibition of beer volatiles formation by carbon dioxide pressure. J the American Society of Brewing Chemists 35:35–40. 10.1094/ASBCJ-35-0035.

[8] Vrieling AM. 1978. Agitated fermentation in high fermentors. Eur Brew Conv Monograph 5:135–145.

[9] Shen HY, De Schrijver S, Moonjai N, Verstrepen KJ, Delvaux F, Delvaux FR. 2004. Effects of CO2 on the formation of flavour volatiles during fermentation with immobilised brewer's yeast. Appl Microbiol Biotechnol 64:636–643. 10.1007/s00253-003-1523-0. 14676983

[10] Jones RP, Greenfield PF. 1982. Effect of carbon dioxide on yeast growth and fermentation. Enzyme Microb Technol 4:210–223. 10.1016/0141-0229(82)90034-5.

[11] Hodgson JA, King AT, Moir M. 1995. Effects of pressure and nitrogen sparging on flavor development during fermentation, p 411–418, 25th Eur Brew Conv Proc, vol 25, Brussels, Belgium.

[12] Verstrepen KJ, Derdelinckx G, Dufour J, Winderickx J, Thevelein JM, Pretorius IS, Delvaux FR. 2003. Flavor-active esters: adding fruitiness to beer. J Biosci Bioeng 96:110–118. 10.1016/S1389-1723(03)90112-5. 16233495

[13] Verstrepen KJ, Van Laere SD, Vanderhaegen BM, Derdelinckx G, Dufour JP, Pretorius IS, Winderickx J, Thevelein JM, Delvaux FR. 2003. Expression levels of the yeast alcohol acetyltransferase genes *ATF1*, *Lg*-*ATF1*, and *ATF2* control the formation of a broad range of volatile esters. Appl Environ Microbiol 69:5228–5237. 10.1128/AEM.69.9.5228-5237.2003. 12957907 PMC194970

[14] Saerens SM, Delvaux FR, Verstrepen KJ, Thevelein JM. 2010. Production and biological function of volatile esters in *Saccharomyces cerevisiae*. Microb Biotechnol 3:165–177. 10.1111/j.1751-7915.2009.00106.x. 21255318 PMC3836583

[15] Verstrepen KJ, Moonjai N, Bauer FF, Derdelinckx G, Dufour JP, Winderickx J, Thevelein JM, Pretorius IS, Delvaux FR. 2003. Genetic regulation of ester synthesis in yeast: new facts, insights and implications for the brewer, p 234–248. *In* Smart K (ed), Brewing yeast fermentation performance, 2nd ed Oxford, Wiley-Blackwell, Hoboken, New Jersey.

[16] Fujiwara D, Kobayashi O, Yoshimoto H, Harashima S, Tamai Y. 1999. Molecular mechanism of the multiple regulation of the *Saccharomyces cerevisiae ATF1* gene encoding alcohol acetyltransferase. Yeast 15:1183–1197. 10.1002/(SICI)1097-0061(19990915)15:12<1183::AID-YEA444>3.0.CO;2-J. 10487921

[17] Lieb JD, Liu X, Botstein D, Brown PO. 2001. Promoter-specific binding of Rap1 revealed by genome-wide maps of protein-DNA association. Nat Genet 28:327–334. 10.1038/ng569. 11455386

[18] Trindade de Carvalho B, Holt S, Souffriau B, Lopes Brandao R, Foulquie-Moreno MR, Thevelein JM. 2017. Identification of novel alleles conferring superior production of rose flavor phenylethyl acetate using polygenic analysis in yeast. mBio 8:e01173-17. 10.1128/mBio.01173-17. 29114020 PMC5676035

[19] Kunz J, Henriquez R, Schneider U, Deuter-Reinhard M, Movva NR, Hall MN. 1993. Target of rapamycin in yeast, TOR2, is an essential phosphatidylinositol kinase homolog required for G1 progression. Cell 73:585–596. 10.1016/0092-8674(93)90144-f. 8387896

[20] Conrad M, Schothorst J, Kankipati HN, Van Zeebroeck G, Rubio-Texeira M, Thevelein JM. 2014. Nutrient sensing and signaling in the yeast *Saccharomyces cerevisiae*. FEMS Microbiol Rev 38:254–299. 10.1111/1574-6976.12065. 24483210 PMC4238866

[21] Verstrepen K, Derdelinckx G, Dufour J, Winderickx J, Pretorius I, Thevelein J, Delvaux F. 2003. The alcohol acetyl transferase gene is a target of the cAMP/PKA and FGM nutrient-signalling pathways. FEMS Yeast Res 4:285–296. 10.1016/S1567-1356(03)00166-1. 14654433

[22] Garcia-Gonzalez L, Geeraerd AH, Spilimbergo S, Elst K, Van Ginneken L, Debevere J, Van Impe JF, Devlieghere F. 2007. High pressure carbon dioxide inactivation of microorganisms in foods: the past, the present and the future. Int J Food Microbiol 117:1–28. 10.1016/j.ijfoodmicro.2007.02.018. 17475355

[23] McIntyre M, McNeil B. 1998. Morphogenetic and biochemical effects of dissolved carbon dioxide on filamentous fungi in submerged cultivation. Appl Microbiol Biotechnol 50:291–298. 10.1007/s002530051293. 9802213

[24] Aguilera J, Petit T, de Winde JH, Pronk JT. 2005. Physiological and genome-wide transcriptional responses of *Saccharomyces cerevisiae* to high carbon dioxide concentrations. FEMS Yeast Res 5:579–593. 10.1016/j.femsyr.2004.09.009. 15780657

[25] Slaughter JC, Flint PWN, Kular KS. 1987. The effect of CO2 on the adsorption of amino acids from malt extract by *Saccharomyces cerevisiae*. FEMS Microbiol Lett 40:239–243. 10.1111/j.1574-6968.1987.tb02032.x.

[26] Pais TM, Foulquie-Moreno MR, Thevelein JM. 2014. QTL mapping by pooled-segregant whole-genome sequencing in yeast. Methods Mol Biol 1152:251–266. 10.1007/978-1-4939-0563-8_15. 24744038

[27] Swinnen S, Thevelein JM, Nevoigt E. 2012. Genetic mapping of quantitative phenotypic traits in *Saccharomyces cerevisiae*. FEMS Yeast Res 12:215–227. 10.1111/j.1567-1364.2011.00777.x. 22150948

[28] Liti G, Louis EJ. 2012. Advances in quantitative trait analysis in yeast. PLoS Genet 8:e1002912. 10.1371/journal.pgen.1002912. 22916041 PMC3420948

[29] Steyer D, Ambroset C, Brion C, Claudel P, Delobel P, Sanchez I, Erny C, Blondin B, Karst F, Legras JL. 2012. QTL mapping of the production of wine aroma compounds by yeast. BMC Genomics 13:573. 10.1186/1471-2164-13-573. 23110365 PMC3575298

[30] Eder M, Sanchez I, Brice C, Camarasa C, Legras JL, Dequin S. 2018. QTL mapping of volatile compound production in Saccharomyces cerevisiae during alcoholic fermentation. BMC Genomics 19:166. 10.1186/s12864-018-4562-8. 29490607 PMC5831830

[31] Abt TD, Souffriau B, Foulquié-Moreno MR, Duitama J, Thevelein JM. 2016. Genomic saturation mutagenesis and polygenic analysis identify novel yeast genes affecting ethyl acetate production, a non-selectable polygenic trait. Microb Cell 3:159–175. 10.15698/mic2016.04.491. 28357348 PMC5349090

[32] Roncoroni M, Santiago M, Hooks DO, Moroney S, Harsch MJ, Lee SA, Richards KD, Nicolau L, Gardner RC. 2011. The yeast *IRC7* gene encodes a beta-lyase responsible for production of the varietal thiol 4-mercapto-4-methylpentan-2-one in wine. Food Microbiol 28:926–935. 10.1016/j.fm.2011.01.002. 21569935

[33] Marullo P, Aigle M, Bely M, Masneuf-Pomarede I, Durrens P, Dubourdieu D, Yvert G. 2007. Single QTL mapping and nucleotide-level resolution of a physiologic trait in wine *Saccharomyces cerevisiae* strains. FEMS Yeast Res 7:941–952. 10.1111/j.1567-1364.2007.00252.x. 17537182

[34] Noble J, Sanchez I, Blondin B. 2015. Identification of new *Saccharomyces cerevisiae* variants of the *MET2* and *SKP2* genes controlling the sulfur assimilation pathway and the production of undesirable sulfur compounds during alcoholic fermentation. Microb Cell Fact 14:68. 10.1186/s12934-015-0245-1. 25947166 PMC4432976

[35] Holt S, Trindade de Carvalho B, Foulquie-Moreno MR, Thevelein JM. 2018. Polygenic analysis in absence of major effector *ATF1* unveils novel components in yeast flavor ester biosynthesis. mBio 9:01279–18. 10.1128/mBio.01279-18. PMC6113618 30154260

[36] Yang Y, Foulquie-Moreno MR, Clement L, Erdei E, Tanghe A, Schaerlaekens K, Dumortier F, Thevelein JM. 2013. QTL analysis of high thermotolerance with superior and downgraded parental yeast strains reveals new minor QTLs and converges on novel causative alleles involved in RNA processing. PLoS Genet 9:e1003693. 10.1371/journal.pgen.1003693. 23966873 PMC3744412

[37] Farboud B, Meyer BJ. 2015. Dramatic enhancement of genome editing by CRISPR/Cas9 through improved guide RNA design. Genetics 199:959–971. 10.1534/genetics.115.175166. 25695951 PMC4391549

[38] Libkind D, Hittinger CT, Valerio E, Goncalves C, Dover J, Johnston M, Goncalves P, Sampaio JP. 2011. Microbe domestication and the identification of the wild genetic stock of lager-brewing yeast. Proc Natl Acad Sci USA 108:14539–14544. 10.1073/pnas.1105430108. 21873232 PMC3167505

[39] Wendland J. 2014. Lager yeast comes of age. Eukaryot Cell 13:1256–1265. 10.1128/EC.00134-14. 25084862 PMC4187645

[40] Mason AB, Dufour J. 2000. Alcohol acetyltransferases and the significance of ester synthesis in yeast. Yeast 16:1287–1298. 10.1002/1097-0061(200010)16:14<1287::AID-YEA613>3.0.CO;2-I. 11015726

[41] Zacchi LF, Gomez-Raja J, Davis DA. 2010. Mds3 regulates morphogenesis in *Candida albicans* through the TOR pathway. Mol Cell Biol 30:3695–3710. 10.1128/MCB.01540-09. 20457806 PMC2897559

[42] Wenger JW, Piotrowski J, Nagarajan S, Chiotti K, Sherlock G, Rosenzweig F. 2011. Hunger artists: yeast adapted to carbon limitation show trade-offs under carbon sufficiency. PLoS Genet 7:e1002202. 10.1371/journal.pgen.1002202. 21829391 PMC3150441

[43] Brice C, Sanchez I, Bigey F, Legras JL, Blondin B. 2014. A genetic approach of wine yeast fermentation capacity in nitrogen-starvation reveals the key role of nitrogen signaling. BMC Genomics 15:495. 10.1186/1471-2164-15-495. 24947828 PMC4073503

[44] Davis DA, Bruno VM, Loza L, Filler SG, Mitchell AP. 2002. *Candida albicans* Mds3p, a conserved regulator of pH responses and virulence identified through insertional mutagenesis. Genetics 162:1573–1581. 10.1093/genetics/162.4.1573. 12524333 PMC1462392

[45] Thompson JR, Register E, Curotto J, Kurtz M, Kelly R. 1998. An improved protocol for the preparation of yeast cells for transformation by electroporation. Yeast 14:565–571. 10.1002/(SICI)1097-0061(19980430)14:6<565::AID-YEA251>3.0.CO;2-B. 9605506

[46] Duitama J, Quintero JC, Cruz DF, Quintero C, Hubmann G, Foulquie-Moreno MR, Verstrepen KJ, Thevelein JM, Tohme J. 2014. An integrated framework for discovery and genotyping of genomic variants from high-throughput sequencing experiments. Nucleic Acids Res 42:e44. 10.1093/nar/gkt1381. 24413664 PMC3973327

[47] Edwards MD, Gifford DK. 2012. High-resolution genetic mapping with pooled sequencing. BMC Bioinformatics 13 Suppl 6:S8. 10.1186/1471-2105-13-S6-S8. PMC3358661 22537047

[48] DiCarlo JE, Norville JE, Mali P, Rios X, Aach J, Church GM. 2013. Genome engineering in *Saccharomyces cerevisiae* using CRISPR-Cas systems. Nucleic Acids Res 41:4336–4343. 10.1093/nar/gkt135. 23460208 PMC3627607

[49] Stovicek V, Borodina I, Forster J. 2015. CRISPR–Cas system enables fast and simple genome editing of industrial *Saccharomyces cerevisiae* strains. Metab Eng Commun 2:13–22. 10.1016/j.meteno.2015.03.001. 34150504 PMC8193243

[50] Ryan OW, Skerker JM, Maurer MJ, Li X, Tsai JC, Poddar S, Lee ME, DeLoache W, Dueber JE, Arkin AP, Cate JH. 2014. Selection of chromosomal DNA libraries using a multiplex CRISPR system. Elife 3:e03703. 10.7554/eLife.03703. PMC4161972 25139909

[51] Gorter de Vries AR, de Groot PA, van den Broek M, Daran JG. 2017. CRISPR-Cas9 mediated gene deletions in lager yeast *Saccharomyces pastorianus*. Microb Cell Fact 16:222. 10.1186/s12934-017-0835-1. 29207996 PMC5718131

[52] Sherman F, Hicks J. 1991. Micromanipulation and dissection of asci. Methods Enzymol 194:21–37. 10.1016/0076-6879(91)94005-w. 2005789

[53] Huxley C, Green ED, Dunham I. 1990. Rapid assessment of *S. cerevisiae* mating type by PCR. Trends Genet 6:236. 10.1016/0168-9525(90)90190-h. 2238077

[54] Hubmann G, Mathe L, Foulquie-Moreno MR, Duitama J, Nevoigt E, Thevelein JM. 2013. Identification of multiple interacting alleles conferring low glycerol and high ethanol yield in Saccharomyces cerevisiae ethanolic fermentation. Biotechnol Biofuels 6:87. 10.1186/1754-6834-6-87. 23759206 PMC3687583

