# Supplementary material for: Polygenic Analysis of Tolerance to Carbon Dioxide Inhibition of Isoamyl Acetate “Banana” Flavor Production in Yeast Reveals MDS3 as Major Causative Gene
Source: Appl Environ Microbiol. 2022 Sep 8;88(18):e00814-22. doi: 10.1128/aem.00814-22 (PMC9499027; doi:10.1128/aem.00814-22)
Supplement: Supplemental file 1 — Supplemental material. Download aem.00814-22-s0001.pdf, PDF file, 0.7 MB [file aem.00814-22-s0001.pdf]

Polygenic analysis of tolerance to carbon dioxide inhibition of isoamyl acetate "banana" flavor production in yeast reveals *MDS3* as major causative gene

Ben Souffriau[1,2,3,#], Sylvester Holt[1,2,#], Arne Hagman[1,2,#], Stijn De Graeve[1,2], Philippe Malcorps[3], Maria R. Foulquié-Moreno[1,2*] and Johan M. Thevelein[1,2,*]

Supplementary materials

Supplementary table 1. Statistical comparison of flavor compound levels produced in CO_2 pressurized fermentations by the wild-type ER7A *MDS3*^{WT} strain and strains containing missense mutations in the *MDS3* gene. Fermentations were carried out in triplicate with malt extract media and 0.65 bar CO_2 overpressure. Metabolites were measured with GC-FID. P-values from unpaired t-tests with Holm-Sidak correction for multiple testing, were obtained by comparing the values of the reference strain ER7A with those of a strain with either re-insertion of the wild-type *MDS3* allele or an *MDS3* allele with a combination of the missense SNPs C305T (T102M), T2171C (F724S), and A3229G (I1077V), or the complete *MDS3* allele from Seg.63. The Seg.63 superior haploid strain was included as a positive control. Compounds are indicated with abbreviations as follows: AAld, Acetaldehyde; IbAlc, Isobutanol; IaAlc, Isoamyl alcohol; PhAlc, Phenyl ethanol; EAc, Ethyl acetate; IbAc, Isobutyl acetate; IAc, Isoamyl acetate; PhAc, Phenylethyl acetate; EtHex, Ethyl hexanoate; EtOct, Ethyl octanoate; EtDec, Ethyl decanoate.

Supplementary figure 1. Isoamyl acetate production levels determined in the screening of a yeast strain collection for strains with high isoamyl acetate production. The strains with the highest production level were used for evaluation in fermentations with extra CO_2 pressure.

Supplementary figure 2. Residual maltose level after fermentation for four days in malt extract wort by the 428 segregants isolated from the hybrid diploid Seg.63/ER7A. Of the 428 segregants, 65% showed complete maltose fermentation after four days in malt extract wort in microtiter plates, whereas the remainder showed incomplete fermentation of the maltose.

	AAld	IbAlc	IaAlc	PhAlc	EAc	IbAc	IaAc	PhAc	EtHex	EtOct	EtDec
ER7A *MDS3*^{WT}	3,83±0,29	21,6±3,0	86,1±9,9	73,8±3,7	25,6±1,8	0,07±0,01	1,43±0,10	2,63±0,61	0,53±0,03	0,31±0,04	0,23±0,03
ER7A *MDS3*^{C305T}	3,36±1,16 (ns)	19,0±1,5 (ns)	79,8±9,8 (ns)	67,1±23,7 (ns)	23,6±0,5 (ns)	0,07±0,00 (ns)	1,66±0,14 (ns)	2,91±0,62 (ns)	0,61±0,01 (ns)	0,35±0,06 (ns)	0,18±0,08 (ns)
ER7A *MDS3*^{T2171C}	7,79±1,97 (ns)	24,0±1,9 (ns)	95,3±6,6 (ns)	88,1±7,6 (ns)	25,6±1,4 (ns)	0,18±0,01 (***)	4,06±0,18 (***)	4,11±1,93 (ns)	0,60±0,06 (ns)	0,48±0,01 (*)	0,42±0,12 (ns)
ER7A *MDS3*^{A3229G}	4,06±0,35 (ns)	20,3±2,4 (ns)	81,4±12,0 (ns)	61,4±6,0 (ns)	23,9±2,0 (ns)	0,05±0,01 (ns)	1,26±0,15 (ns)	1,94±0,13 (ns)	0,50±0,06 (ns)	0,30±0,03 (ns)	0,17±0,01 (ns)
ER7A *MDS3*$^{C305T, T2171C}$	5,43±0,66 (ns)	21,3±0,6 (ns)	86,7±2,4 (ns)	72,7±1,2 (ns)	23,3±1,8 (ns)	0,16±0,01 (**)	3,95±0,18 (***)	2,46±0,55 (ns)	0,66±0,04 (ns)	0,46±0,02 (*)	0,35±0,11 (ns)
ER7A *MDS3*$^{C305T, A3229G}$	3,32±0,95 (ns)	18,6±2,9 (ns)	74,0±10,2 (ns)	56,3±10,8 (ns)	22,7±1,1 (ns)	0,06±0,00 (ns)	1,46±0,06 (ns)	2,53±0,19 (ns)	0,51±0,06 (ns)	0,32±0,01 (ns)	0,22±0,01 (ns)
ER7A *MDS3*$^{T2171C, A3229G}$	6,48±1,68 (ns)	24,5±2,2 (ns)	87,1±10,7 (ns)	77,5±36,3 (ns)	22,6±3,4 (ns)	0,17±0,04 (ns)	3,50±0,53 (*)	3,28±1,22 (ns)	0,49±0,09 (ns)	0,41±0,06 (ns)	0,32±0,05 (ns)
ER7A *MDS3*$^{C305T, 2171C, A3229G}$	5,85±0,49 (*)	21,0±0,9 (ns)	82,1±2,7 (ns)	69,4±5,9 (ns)	22,5±1,0 (ns)	0,15±0,01 (***)	3,44±0,08 (***)	2,22±0,30 (ns)	0,49±0,05 (ns)	0,40±0,01 (ns)	0,31±0,04 (ns)
ER7A *MDS3*$^{Seg.63}$	6,52±2,53 (ns)	20,1±2,9 (ns)	76,6±8,3 (ns)	76,5±7,7 (ns)	20,1±2,1 (ns)	0,15±0,02 (*)	3,24±0,38 (*)	2,31±0,38 (ns)	0,46±0,07 (ns)	0,36±0,03 (ns)	0,28±0,06 (ns)
Seg. 63	4,20±0,70 (ns)	10,6±1,4 (*)	64,3±5,1 (ns)	73,2±7,2 (ns)	36,1±1,9 (*)	0,11±0,01 (*)	4,18±0,35 (**)	2,80±0,30 (ns)	0,37±0,07 (ns)	0,40±0,03 (ns)	0,11±0,03 (ns)

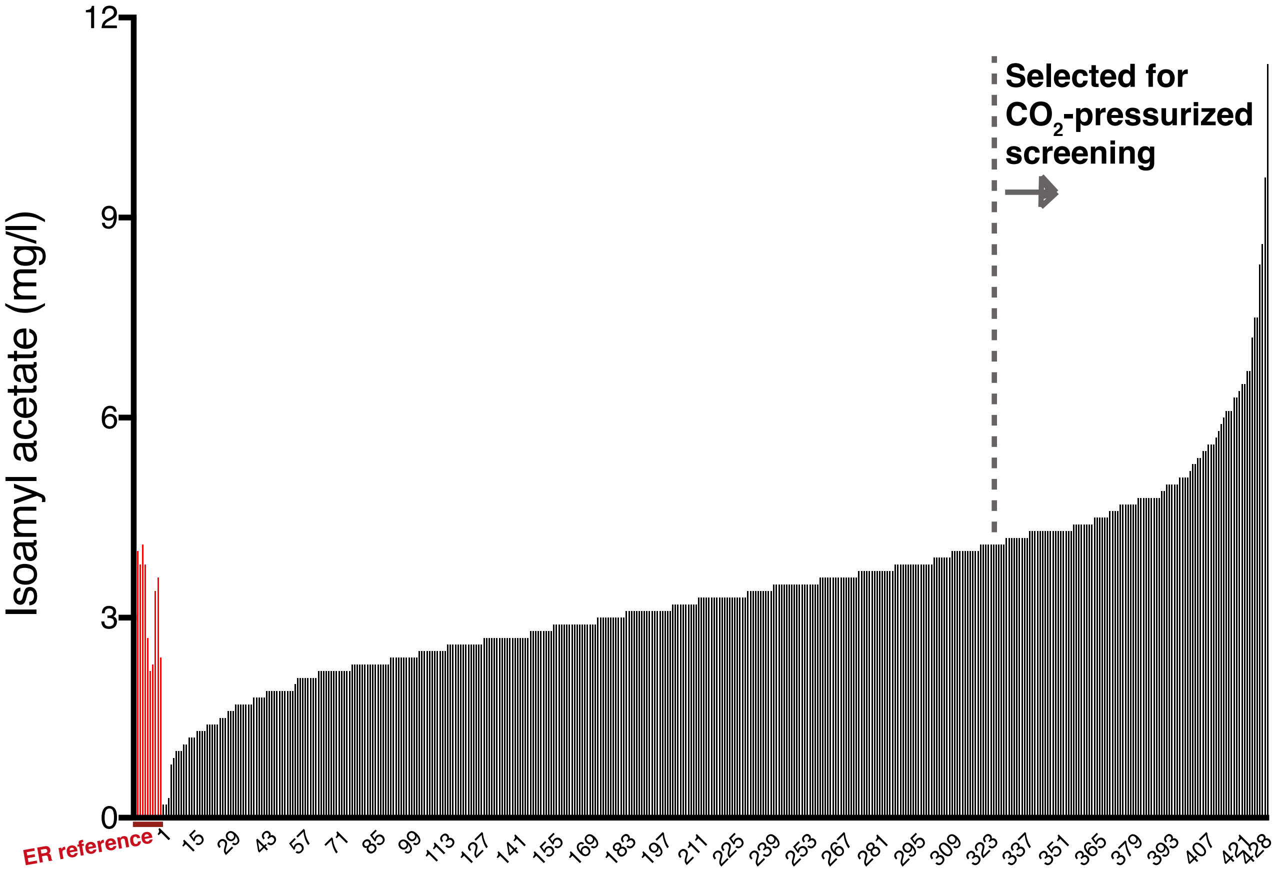
Isoamyl acetate (mg/l)
0
3
6
9
12
Selected for
CO_2-pressurized
screening
ER reference
1
15
29
43
57
71
85
99
113
127
141
155
169
183
197
211
225
239
253
267
281
295
309
323
337
351
365
379
393
407
421
428

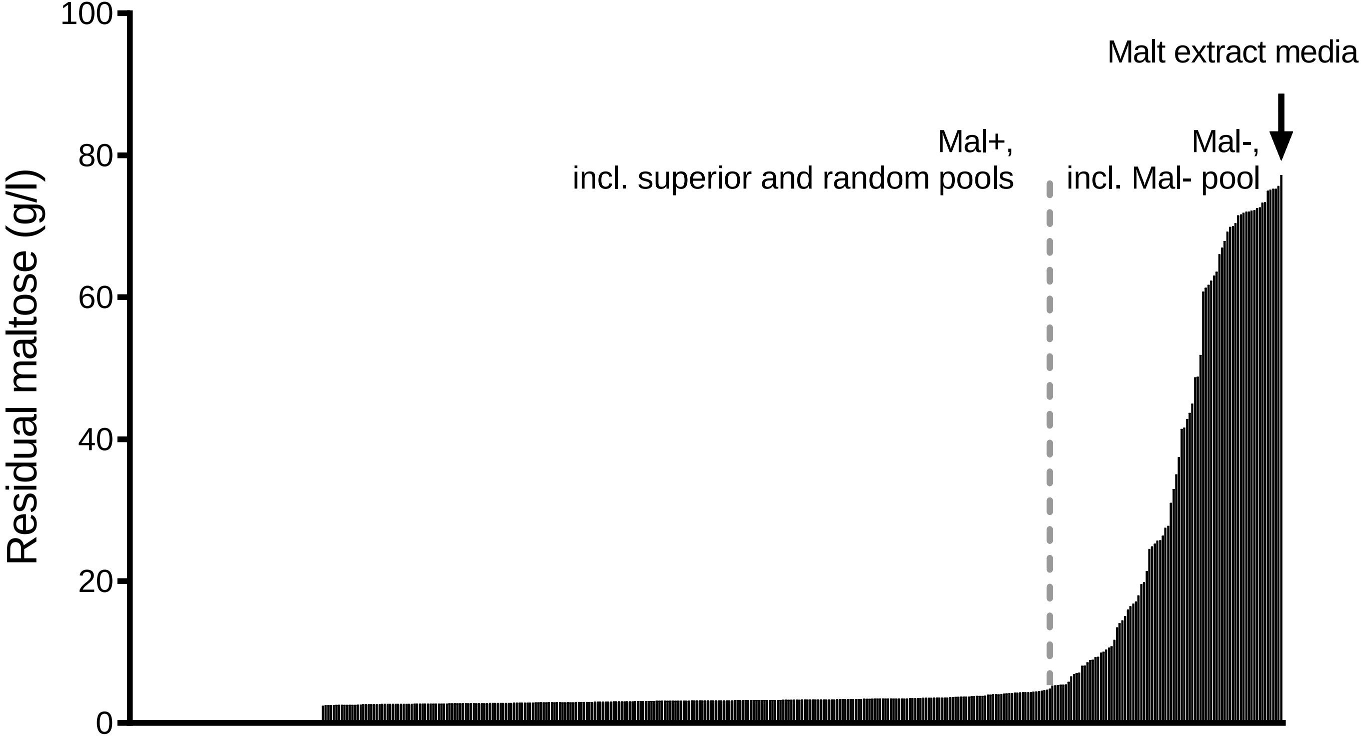
Malt extract media
Mal+,
incl. superior and random pools
Mal-,
incl. Mal- pool
Residual maltose (g/l)
100
80
60
40
20
0
